# Very High Bit Rate Near-Field Communication with Low-Interference Coils and Digital Single-Bit Sampling Transceivers for Biomedical Sensor Systems

**DOI:** 10.3390/s20216025

**Published:** 2020-10-23

**Authors:** Sebastian Stoecklin, Elias Rosch, Adnan Yousaf, Leonhard Reindl

**Affiliations:** Laboratory for Electrical Instrumentation, Department of Microsystems Engineering, University of Freiburg, 79110 Freiburg, Germany; eliasrosch@googlemail.com (E.R.); adnan.yousaf@gmail.com (A.Y.); leonhard.reindl@imtek.uni-freiburg.de (L.R.)

**Keywords:** near-field communication, very high bit rate, low-power systems, figure-8 coils, radio-frequency transceivers, single-bit sampling receiver, phase-shift keying, bit error rate

## Abstract

The evolution of microelectronics increased the information acquired by today’s biomedical sensor systems to an extent where the capacity of low-power communication interfaces becomes one of the central bottlenecks. Hence, this paper mathematically analyzes and experimentally verifies novel coil and transceiver topologies for near-field communication interfaces, which simultaneously allow for high data transfer rates, low power consumption, and reduced interference to nearby wireless power transfer interfaces. Data coil design is focused on presenting two particular topologies which provide sufficient coupling between a reader and a wireless sensor system, but do not couple to an energy coil situated on the same substrate, severely reducing interference between wireless data and energy transfer interfaces. A novel transceiver design combines the approaches of a minimalistic analog front-end with a fully digital single-bit sampling demodulator, in which rectangular binary signals are processed by simple digital circuits instead of sinusoidal signals being conditioned by complex analog mixers and subsequent multi-bit analog-to-digital converters. The concepts are implemented using an analog interface in discrete circuit technology and a commercial low-power field-programmable gate array, yielding a transceiver which supports data rates of up to 6.78 MBit/s with an energy consumption of just 646 pJ/bit in transmitting mode and of 364 pJ/bit in receiving mode at a bit error rate of $2\times 10^{-7}$, being 10 times more energy efficient than any commercial NFC interface and fully implementable without any custom CMOS technology.

## 1. Introduction

Near-field communication (NFC) has proven to be a reliable data transfer technology in a series of biomedical implants and devices: the communication interface, based on inductive coupling of two coils, is hereby complementing the actual sensor front-end by establishing the connection to the corresponding data processing unit. Among others, inductive communication technologies have been applied in biomedical sensor systems to monitor physiological parameters such as heart rate, blood pressure, or gastrointestinal activity [1,2], to analyze biochemical fluids [3,4], to improve hearing in cochlear implants [5], to reestablish vision in retinal implants [6], and to analyze and record neural signals in the context of brain–computer interfaces [7].

Improved methods and materials in electrical and biomedical engineering are recently driving the need for near-field interfaces with very high data rates in the range of several megabits per second: on the one hand, sensor systems store data over extended periods of time, which need to be acquired in regular time intervals. Here, a fast communication speed to extract extended datasets will increase convenience of patients and physicians. On the other hand, there are technical systems in which data are not only sampled with high resolution, but simultaneously at hundreds of channels. Examples of such systems are neural implants, which record the electrical signals of the brain’s neurons with high spatial and temporal resolution [8,9], in order to detect seizures of epilepsy or Parkinson’s disease, which is useful in understanding the electrochemical processes and in mitigating the symptoms by subsequent electrical stimulation. Taking the highly integrated silicon neural probes of [9] as an example, the raw data output stream from implant to the reader unit of such a device using 144 channels, a sampling rate of 20 kHz and a resolution of 11 bit per channel can be as high as 32 MBit/s. This certainly requests a powerful extracorporeal data processing unit, which must be connected wirelessly in order to avoid any source of inflammation emerging from wired connections. The concepts of closed-loop neural interfaces even require fast bidirectional data communication, as they apply high-resolution and multi-channel electrical stimulation controlled by the extracorporeal data processing unit in order to suppress the symptoms of detected neural seizures [10]. Hence, very high bit rate NFC systems are a key technology to enable these applications.

### 1.1. Physical Background

**Figure 1:**
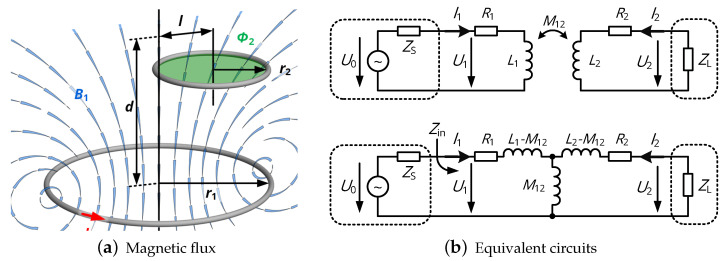
Physical and electrical representations of a near-field system.

In general, near-field communication is based on the principle of inductive coupling, where a sinusoidal current of amplitude $I_{1}$ and angular frequency ω is injected into a primary coil and thus generates a magnetic field of flux density $B_{1}$. In a secondary coil spanning the area $S_{2}$, $B_{1}$ creates a magnetic flux (see Figure 1a)

(1) $$\begin{array}{c} \Phi_{2}=\underset{S_{2}}{\;\int \int \;}{\vec{B}}_{1}\left(\vec{r}\right)\;d{\vec{S}}_{2}, \end{array}$$

which induces a voltage $U_{2}$ according to Faraday’s law:

(2) $$\begin{array}{c} U_{2}=-\frac{d\Phi_{2}}{dt}=-\underset{S_{2}}{\;\int \int \;}j\omega {\vec{B}}_{1}\left(\vec{r}\right)\;d{\vec{S}}_{2}=j\omega M_{12}I_{1}. \end{array}$$

Here, the ratio of magnetic flux $\Phi_{2}$ and primary current $I_{1}$ is defined as the mutual inductance

(3) $$\begin{array}{c} M_{12}=\frac{\Phi_{2}}{I_{1}} \end{array}$$

and is a measure of the coupling strength of primary and secondary coil. Moreover, the system is also characterized by the inductances $L_{i}$ and the loss resistances $R_{i}$ of the coils (with index i∈{1,2} denoting the coil number). The primary coil is driven by a voltage source with source impedance $Z_{S}$, the secondary coil is loaded with a circuit of impedance $Z_{L}$, resulting in the equivalent circuit of Figure 1b. In order to present all physical aspects being relevant to near-field data transfer systems, we need to account for the loaded input impedance, which is present at the terminals of the primary coil and is composed of the primary’s self-impedance $Z_{\mathrm{self}}$ and the reflected impedance $Z_{r}$ representing the power transfer to the secondary coil:

(4) $$\begin{array}{c} Z_{\mathrm{in}}=Z_{\mathrm{self}}+Z_{r}=R_{1}+j\omega L_{1}+\frac{\omega^{2}M_{12}^{2}}{j\omega L_{2}+R_{2}+Z_{L}}. \end{array}$$

With the given physical background, two data transfer mechanisms can be established: The first category is classified as *load modulation* or *load shift keying* (LSK): The primary device continuously provides RF energy by a sinusoidal input voltage $U_{0}$ with constant amplitude, while the secondary device transmits binary data by changing its load impedance $Z_{L}$: By enabling or disabling additional resistive or capacitive branches, binary information (’0’ or ’1’) is encoded. Consequently, the input impedance is modified according to (4), so that a change in the logic level reflects to a change in the envelope signal of $U_{1}$. The second category of data transmission requires that every participating communication device *actively generates a modulated carrier signal* by modifying the properties of its source voltage $U_{0}$ (either by amplitude, phase or frequency modulation), translating to a change in the secondary’s output voltage $U_{2}$ (also modulated w.r.t. amplitude, phase or frequency) which is provided to the corresponding demodulation stage.

### 1.2. Preliminary Work

Up to now, standardized devices in radio frequency identification (RFID) and near-field communication (NFC) are limited to data rates of up to 848 kBit/s when using load modulation. Commercial devices implementing custom protocols with active signal generation achieve up to 3.4 MBit/s, but at the cost of extended energy consumption (approx. 20 mW in receive mode) [11].

Due to the lack of commercial NFC technologies complementing the presented biomedical sensor systems with high data throughput, a series of research efforts have been taken to yield solutions with both high communication speed and low energy consumption:

Mandal et al. were among the first to increase the data rate of load modulation systems: a near-field interface without simultaneous power transfer capabilities was implemented by custom CMOS circuits operating at a carrier frequency of 25 MHz. The implant circuits incorporated a simple switch-based transmitter for impedance modulation with a power consumption of 0.1 mW. At the reader side, a custom PLL-based clock and data recovery unit extracted the transmitted data. With these characteristics, an uplink data rate (from implant to reader unit) of 2.8 MBit/s at a bit error rate (BER) of smaller than $10^{-6}$ was demonstrated [12]. Remaining limitations are the relatively small communication distance as a consequence of the load modulation principle, as well as the susceptibility to interference from an additional energy link.

Active signal generation concepts were improved by Simard et al., who used custom CMOS circuits with a modified Costas loop receiver to implement a 4-PSK modulation on the inductive link and an arrangement of six coils in total to allow for simultaneous energy and data transfer. The data link was capable of achieving 4.18 MBit/s with a BER smaller than $10^{-5}$ at a carrier frequency of 13.56 MHz [13]. Complementing the energy interface, which was realized in the form of two planar spiral coils, separated data coils are available for both uplink and downlink. The presented data coil topologies either imply a large spatial separation from the energy coil or require the coil axes to be tilted by 90°, in order to minimize interference of energy and data link. Still, the decoupling of energy and data interface is only valid for perfect alignment of implant and reader.

Along with a proposed very high bit rate (VHBR) extension to the ISO-14443 standard, Van de Beek et al. from *NXP Semiconductors* developed a 16-PSK receiver based on a time-to-digital converter, supporting a data rate of up to 13.56 MBit/s with a BER below $2.4\times 10^{-4}$ [14]. The analog front-end, including a phased-locked loop and a time-to-digital converter, consumes 0.1 mW; this excludes the data processing and symbol recovery, which was outsourced to an FPGA circuit of unknown energy consumption.

Another concept frequently applied in literature is that of pulse-harmonic modulation (PHM). Here, a primary LC resonator is excited by pulses encoding a digital ’1’, causing a decaying oscillation in both the primary and secondary resonator. The concept was adapted e.g., by Kiani et al., who established a 20 MBit/s inductive link at an LC resonance frequency of 66 MHz [15]. To adapt PHM for simultaneous wireless power transfer, the authors investigated a 4-coil system in which the pulses evoked by data communication circuit shift the zero-crossings of the 13.56 MHz energy carrier [16]. With this so-called pulse delay modulation scheme, a data rate of 13.56 MBit/s at a BER of $4.7\times 10^{-7}$ was reported, which is valid for the downlink from reader to implant and only for certain coil alignments and power levels of the wireless energy transmission. In the field of coil design, the workgroup published a design of a data transfer interface with two figure-8 coils [17] to minimize interaction and interference; its relatively complex shape and the full assignment of two conductive layers of the substrate might hinder its general application.

Schormans et al. refined the PHM concept by incorporating an additional negative pulse to decrease the duration of the ringing oscillation, achieving 50 MBit/s with structures being resonant at 205 MHz at an impressive bit error rate of $4.5\times 10^{-10}$ [18]. The compatibility with wireless power transfer remains unknown, even though non-overlapping spectral components of data and energy signals were proven.

### 1.3. Scope and Structure of This Work

While these recent concepts allow for a high data rate communication at suitable power levels, there are two general limitations: first of all, the communication systems are either highly dependent on the properties of the parallel wireless power transfer interface or generally incompatible with such an additional near-field interface. Secondly, the implementations all rely on custom CMOS designs, so that their applicability and adaptability are not directly assured. It is therefore the objective of this work

1. to provide electromagnetic interfaces and analog front-ends that are resilient against the parallel operation of a wireless power transfer interface and
2. to present a minimalistic and mostly digital transceiver architecture that allows for implementing the NFC circuitry with a small footprint, reduced energy consumption and low bit error rate, favorably in one of today’s miniaturized low-power FPGAs with only a few additional off-the-shelf components.

The main methodological steps and circuit blocks to reach these objectives are presented in this paper: first, we will present the core ideas and novel approaches of the overall system, being the baseline for subsequent analysis of the electromagnetic interface (i.e., the data transfer coils and their capacitive tuning), of the analog front-end (including a low-power amplifier and impedance matching/filtering networks) and of the digital modulation and demodulation circuits. The overall system will be investigated both theoretically with respect to bit error rate as well as experimentally, where characteristics on data rate, energy consumption and bit error rate are acquired from prototype circuits and compared to existing solutions.

## 2. System Concept

**Figure 2:**
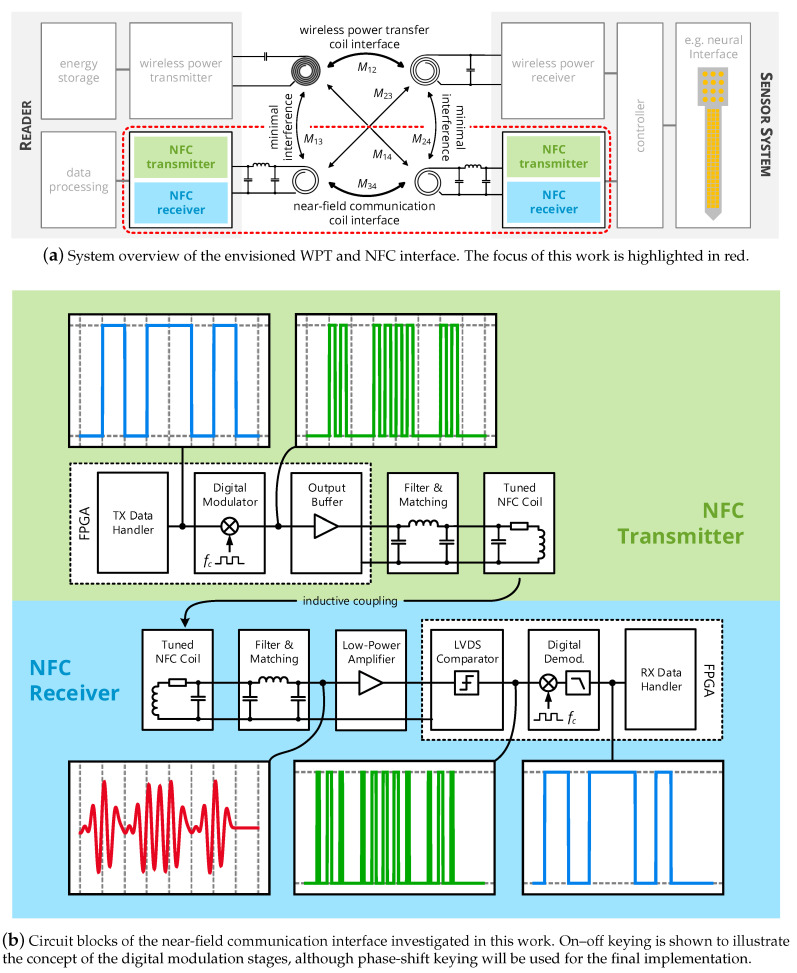
System concept of this work: A separate inductive data communication interface is implemented by two additional coils, which can be subject to an individual optimization. The near-field communication interface is based on an active communication with digital modulators and demodulators, so that data are modulated on a rectangular wave, transmitted over an analog channel and converted back to a rectangular wave by a comparator stage in the receiver, where it gets demodulated by a fully digital circuit.

This work’s concept of a custom near-field interface is illustrated in Figure 2 and incorporates the following particular features:

- Separate coils are used for data and energy transfer. Hence, the data resonators can be optimized for a low quality factor (1 to 10) to realize a high data link bandwidth in the same order of magnitude as the carrier frequency, in line with established theory on the ringout behavior of LC resonators [13,19].
- To avoid interference in the data link and detuning of the energy link, coupling of energy and data coils, even being located on the same substrate, must be avoided by suitable geometrical design of the coils. Hence, novel coil topologies are presented and analyzed.
- Selecting the operational frequency of the data interface to be lower than that of the energy link allows for extracting the data signal with simple low-pass instead of bandpass filters. Moreover, harmonics from the power transfer interface cannot fall into the communication band. In this work, near-field communication will cover the 13.56 MHz industrial scientific and medical (ISM) band, while the 40.68 MHz ISM band is utilized for wireless power transfer.
- Active signal generation is applied, i.e., both implant and reader unit feature the same transceiver unit to realize a symmetric data link with equal communication capabilities for both devices. This enables the application in closed-loop systems with high bandwidth requirements in both communication directions. Moreover, realizing a broadband communication from reader to implant increases channel availability in the given half-duplex system.
- Modulation and demodulation circuits shall be realized as purely digital circuits to enable a compact implementation. This means that the modulated carrier is generated as a rectangular wave in a digital circuit; a digital class-D output stage successively applies the signal to the analog filtering and data coil stages, which extract the modulated sinusoidal signal. In the receiver, the sinusoidal signal is converted back to a rectangular wave by a comparator stage, and is then demodulated by a purely digital circuit. While comparator-based receivers processing rectangular signals [20,21] and single-bit sampling receivers processing time-discretized samples of a rectangular signal [22] were conceived for biomedical near-field communication, the reliability of their custom demodulation stages in terms of the bit error rate is analyzed neither by simulation nor by measurement. Furthermore, their applicability is limited, as they rely on custom CMOS circuits and frequency bands not reserved for industrial, scientific, and medical (ISM) purposes. The unique contribution of this work is to analyze and evaluate a transceiver that adapts the complex and energy-intensive operations of mixing, low-pass filtering and and multi-bit quantization to the domain of single-bit sampling receivers: Low-power digital circuits discretize the analog data signal with respect to amplitude and time and demodulate the samples by applying digital operations of downconversion and filtering. The transceiver shall be implemented based on commercially available and small-scale electronic components, such as recent ultra-low-power field-programmable gate arrays, and adapted to an ISM frequency band to enhance its applicability.

In particular, the objective is to create a near-field communication interface using a 13.56 MHz carrier with a symbol rate of 6.78 MHz, so that only two carrier cycles will encode a single symbol. As the digital receiver will only allow for constant-envelope modulation [23] and as on-off keying has the potential drawback of ringouts still being detected as an active carrier, binary phase-shift keying will be used as a modulation technique. The expected raw data rate is thus 6.78 MBit/s. While a higher data carrier frequency (e.g., 27.12 MHz) could be chosen to theoretically increase the communication bandwidth, it also correlates with a higher sampling rate, implying a higher power consumption, and with a reduced frequency separation of energy and data carrier, increasing interference for the same steepness of the filtering stages.

As far as the system dimensions are considered, miniaturized neural probes are accompanied by coils [24] and circuit boards [25,26,27] measuring up to several centimeters, which is still feasible as they are embedded in upper tissue layers. Consequently, implant coils and prototype circuits for concept evaluation shall be realized with a maximum edge length of 10 mm, i.e., they shall cover a maximum circuit board area of 100 mm${}^{2}$ each.

The appropriate design mechanism of the individual circuit blocks of Figure 2 are detailed in the following, along with the mathematical foundations leading to a suitable dimensioning of all components.

## 3. Resonator Design

### 3.1. Data Coil Design

Decoupling energy and data interface regardless of a close placement of the corresponding coils is the prerequisite to establish a stable and resilient data communication. The energy inductors used in this work are two capacitively segmented planar spiral coils with radii of 15 mm and 5 mm, respectively, which are designed by the authors of this paper in [28] for a high-efficiency wireless power transfer link operating at 40.68 MHz. Due to the capacitive elements included into the traces, the current distribution is uniform along the conductor and parasitic and lossy capacitive displacement currents are limited, so that high-frequency operation in biological tissue is possible without sacrificing efficiency: For coil separation distances of 20 mm, coil efficiency levels of up to 32% are achievable, while the maximum receivable power at the receiver is approximately 30 mW to fall below the regulatory limit of the specific absorption rate (SAR) of 1.6 W/kg.

**Figure 3:**
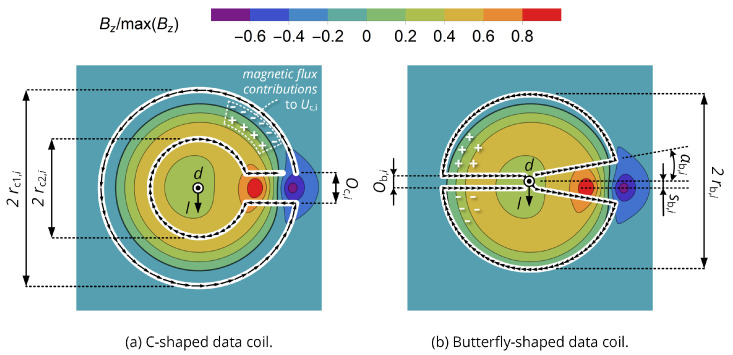
Data coil topologies to minimize mutual inductance to an energy coil on the same substrate and magnetic flux density distribution $B_{z}$ of a planar spiral coil from [28]. By incorporating flux components of opposite sign, the effective flux in the data coil structure can be minimized. Zero coupling can be achieved by appropriate selection of $r_{c2,i}$ and $s_{b,i}$.

Creating a data coil on the same substrate now requires enclosing magnetic flux components of opposite sign, which basically leads to the winding designs shown in Figure 3: Here, the magnetic flux density $B_{z}$ generated by a regular planar spiral energy coil normal to its surface is visualized for a uniform current distribution along its conductor trace and obtained from the numerical evaluation of the Biot–Savart law.

The **C-shaped data coil** can now achieve zero mutual inductance to the energy coil by incorporating flux components of negative sign in the range of the outer winding part and flux components of positive sign along the inner winding part. As the inner winding part is the return line of the current and only the enclosed area contributes to the net flux such as given by (1), the center of the flux distribution does not affect the induced voltage. To include negative flux components, $r_{c1,i}$ must be slightly larger than the radius of the energy coil, while $r_{c2,i}$ is swept in a numerical simulation to yield a design with no coupling to the primary energy coil.

The **butterfly-shaped data coil** follows the principles as elaborated in [17]: Every turn is split into two separate, but axis-symmetric parts with counter-rotating winding direction. If these are situated in a magnetic field of the same symmetry, both parts are subject to the same induced voltage, but, with the opposite sign, so that the overall output voltage and hence mutual inductance is zero. Due to the slightly asymmetric nature of the given energy coils (Archimedean spirals), a small spatial offset $s_{b,i}$ is included to achieve zero coupling. The particular features of the butterfly-shaped coil is that the angular cutout, shown on the right side of Figure 3b, allows for implementing the feeding structure of the energy coil on a two-layer substrate, while the rounded outer contour line limits the size to that of the corresponding energy coil.

It is now to be investigated how well these coil topologies perform in a data link and how they interfere with the second energy coil, which is not located on the same substrate. The notational convention used in the following is that the indices 1 and 2 denote primary and secondary energy coil, 3 and 4 indicate the primary and secondary data coil, respectively. It is therefore of interest to compute $|M_{34}|$ for various coil distances *d* and lateral displacements *l* (see Figure 1a) to spot the regions of limited data coil coupling, as well as the ratio $|M_{14}\left|/\right|M_{34}|$ to obtain the strength of interference from primary energy to secondary data coil and $|M_{23}\left|/\right|M_{34}|$ to analyze interference from secondary energy to primary data coil.

To compute the mutual inductance even for the case of these non-regular coils, the Neumann formula describing $M_{ik}$ based on line integrals along the conductor traces of both coils *i* and *k* (see, e.g., [29]) is approximated by

(5) $$\begin{array}{cc} M_{ik} & =\frac{\mu_{0}}{4\pi }\oint_{l_{i}}\oint_{l_{j}}\frac{d{\vec{r}}_{i}\cdot d{\vec{r}}_{k}}{|{\vec{r}}_{i}-{\vec{r}}_{k}|} \end{array}$$

(6) $$\begin{array}{cc} \; & \approx \frac{\mu_{0}}{4\pi }\sum_{j=1}^{z_{i}-1}\sum_{l=1}^{z_{k}-1}\frac{\left({\vec{r}}_{i(j+1)}-{\vec{r}}_{ij}\right)\cdot \left({\vec{r}}_{k(l+1)}-{\vec{r}}_{kl}\right)}{|{\vec{r}}_{ij}-{\vec{r}}_{kl}|}. \end{array}$$

This means that both coil trajectories are discretized into a series of $z_{i}$ points with coordinates ${\vec{r}}_{ij}$, so that an infinitesimal line element of the Neumann equation becomes a vector between two subsequent trajectory points of a coil (such as indicated by the small black arrows in Figure 3) and that the distance of the line elements to be integrated becomes the distance of the vectors’ end points.

Self-inductance can also be approximated with this discretization by computing the mutual inductance of a coil to itself (i=k); a small offset $|\Delta {\vec{r}}_{o}\left|\ll \right|{\vec{r}}_{ij}-{\vec{r}}_{il}|$ must, however, be included in order to avoid singularities:

(7) $$\begin{array}{c} L_{i}\approx \frac{\mu_{0}}{4\pi }\sum_{j=1}^{z_{i}-1}\sum_{l=1}^{z_{i}-1}\frac{\left({\vec{r}}_{i(j+1)}-{\vec{r}}_{ij}\right)\cdot \left({\vec{r}}_{i(l+1)}-{\vec{r}}_{il}\right)}{|{\vec{r}}_{ij}-{\vec{r}}_{il}+\Delta {\vec{r}}_{o}|}. \end{array}$$

In all simulations of this work, we used 1000 discretization points per turn, so that $z_{i}=1000\times N_{i}$. The coordinates ${\vec{r}}_{ij}$ of the coil trace vectors of Figure 3 were parametrized in a custom program written in *Wolfram Mathematica 12.1*; mutual inductances and self-inductances were then obtained by numerically evaluating the summations in (6) and (7) among all points ${\vec{r}}_{ij}$.

With this numerical approach, both coil topologies were optimized for minimal interference to the on-substrate energy coils by selecting $r_{c2,i}$ or $s_{b,i}$ to yield $M_{13}=M_{24}=0$. In all cases, an outer radius of 17 mm for the primary and 7 mm for the secondary data coil (slightly larger than the energy coils) and three turns (yields inductance values that allow a suitable impedance matching to 100 Ω, see the next section) are assumed. Subsequently, $|M_{34}|$ characterizing the data interface as well as $|M_{14}\left|/\right|M_{34}|$ and $|M_{23}\left|/\right|M_{34}|$, characterizing the interference to the WPT system, are computed from (6) for various distances *d* and lateral misalignments *l* (cf. Figure 3 for coordinates).

**Figure 4:**
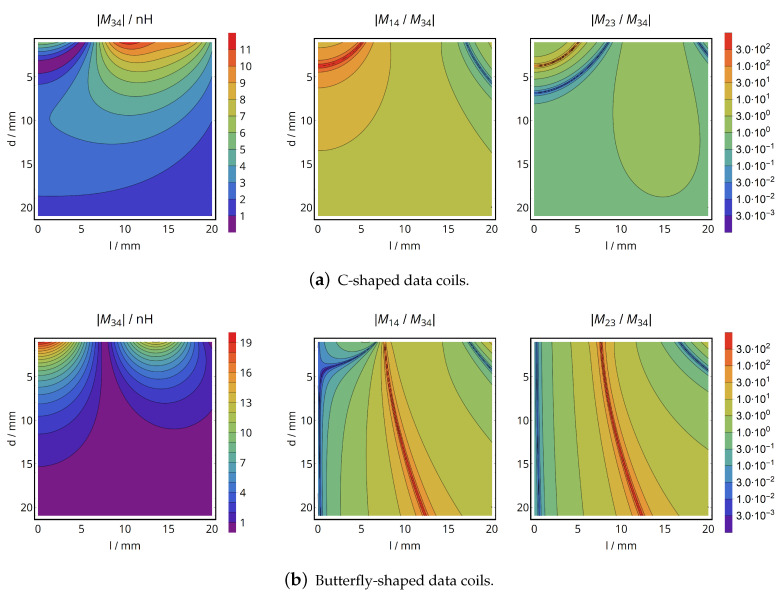
Positional analysis of the data coils’ mutual inductance and the cross-coupling to the energy coils.

The results are shown in Figure 4: A data interface consisting of the C-shaped coils reveals a zero of mutual inductance for a coil separation distance of d=4mm and axial alignment (l=0mm), so that data transfer is strongly limited at this spatial arrangement. The limited mutual inductance in this region also facilitates potential interference from the primary energy coil due to a high ratio of $|M_{14}\left|/\right|M_{34}|$.

When being axially aligned, the butterfly-shaped coils show a monotonic decrease of the data coils’ mutual inductance for an increasing distance and feature a particularly small mutual inductance to the energy coils (low interference), so that they are selected for the system implementation due to these favorable characteristics. Still, it must be noted that the butterfly-shaped coils also exhibit a zero of mutual inductance for lateral displacement, being situated at l≈10mm for the given design.

### 3.2. Data Coil Tuning

Resonant tuning of the data coil is required to provide an output impedance level that allows for a subsequent matching to the receiving amplifier. Here, we attempt to obtain closed-form expressions for the tuning components that provide both impedance matching and fast transient settling.

**Figure 5:**
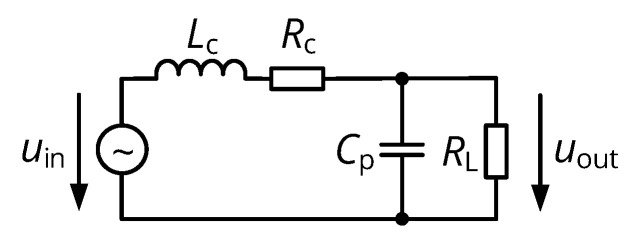
Equivalent circuit of the receiver’s resonator consisting of the data coil $L_{c}$, a series resistance $R_{c}$, a parallel tuning capacitor $C_{p}$, and the load resistance $R_{L}$ representing the receiving amplifier with additional impedance matching circuits.

The basic tuning topology is shown in Figure 5: The receiver coil inductance $L_{c}$ and series resistance $R_{c}$ are complemented by a parallel tuning capacitor $C_{p}$ and a resistance $R_{L}\gg R_{c}$, which represents the input resistance of the subsequent low-pass filter and receiving amplifier. The system is optimized for the case of a large coil separation distance (small $M_{34}$), so that the effect of reflected impedance of (4) can be neglected and the input voltage $u_{\mathrm{in}}$ is determined by Faraday’s law as formulated in (2). Then, the maximum power theorem (see [30], p. 78) requires the impedance resulting from the parallel combination of $C_{p}$ and $R_{L}$ to be the conjugate complex of the coil series impedance:

(8) ReRL1+jω0CpRL=!RcandImRL1+jω0CpRL=!−ω0Lc,

which results in the optimal values of parallel capacitance and load resistance of

(9) $$\begin{array}{cc} C_{p,\mathrm{opt}} & =\frac{L_{c}}{R_{c}^{2}+\omega_{0}^{2}L_{c}^{2}}, \end{array}$$

(10) $$\begin{array}{cc} R_{L,\mathrm{opt}} & =R_{c}+\frac{\omega_{0}^{2}L_{c}^{2}}{R_{c}}. \end{array}$$

Apart from impedance matching, the resonator’s properties must allow for a fast transient response in order to achieve high symbol rates. Consequently, the settling time of the resonator is determined in the following:

First, the frequency behavior of the resonant network is characterized by the transfer function

(11) $$\begin{array}{c} G\left(s\right)=\frac{u_{\mathrm{out}}\left(s\right)}{u_{\mathrm{in}}\left(s\right)}=\frac{R_{L}}{C_{p}L_{c}R_{L}s^{2}+C_{p}R_{c}R_{L}s+L_{c}s+R_{c}+R_{L}}. \end{array}$$

Any symbol transition, either being the activation of the carrier in on-off keying or a phase change in phase-shift keying, is formally represented by an additional sinusoidal input signal $x_{c}\left(t\right)$ adding up to the overall input signal

(12) $$\begin{array}{cc} u_{\mathrm{in}}\left(t\right)=x_{0}\left(t\right)+\Theta \left(t\right)\cdot x_{c}\left(t\right) & =x_{0}\left(t\right)+\Theta \left(t\right)\cdot a_{c}\cdot \sin\left(\omega_{0}t+\phi_{c}\right), \end{array}$$

which is enabled for t>0 by the Heaviside step function Θ(t). This evokes an additional transient output signal $y_{c}\left(t\right)$, which can be obtained from the inverse Laplace transform:

(13) $$\begin{array}{cc} y_{c}\left(t\right) & =L^{-1}\left\{Y_{c}\left(s\right)\right\}\left(t\right)=L^{-1}\left\{G\left(s\right)\cdot X_{c}\left(s\right)\right\}\left(t\right), \end{array}$$

(14) $$\begin{array}{cc} \; & =L^{-1}\left\{G\left(s\right)\cdot a_{c}\cdot \frac{\omega_{0}\cos\phi_{c}+s\sin\phi_{c}}{s^{2}+\omega_{0}^{2}}\right\}\left(t\right), \end{array}$$

(15) $$\begin{array}{cc} \; & =a_{c}\cdot \left(e^{-\alpha t}\left(A_{1}e^{j\beta t}+A_{2}e^{-j\beta t}\right)+A_{3}\cos\left(\omega_{0}t\right)+A_{4}\sin\left(\omega_{0}t\right)\right), \end{array}$$

Here, $A_{i}$ are complex coefficients depending on the equivalent circuit parameters and the excitation frequency $\omega_{0}$, while α and β are analytically given as

(16) $$\begin{array}{cc} \alpha & =\frac{1}{2C_{p}R_{L}}+\frac{R_{c}}{2L_{c}}\overset{\left(9),(10\right)}{=}\frac{R_{c}}{L_{c}}, \end{array}$$

(17) $$\begin{array}{cc} \beta & =\frac{\sqrt{4C_{p}L_{c}R_{L}\left(R_{c}+R_{L}\right)-{\left(C_{p}R_{c}R_{L}+L_{c}\right)}^{2}}}{2C_{p}L_{c}R_{L}}\overset{\left(9),(10\right)}{=}\sqrt{1+{\left(\frac{\omega_{0}L_{c}}{R_{c}}\right)}^{2}}\cdot \frac{R_{c}}{L_{c}}. \end{array}$$

Hence, the steady state solution is determined by the last two terms of (15) and the transient response by its first term, which is a decaying oscillation of angular frequency β∈R and the decay time

(18) $$\begin{array}{cc} \tau =\frac{1}{\alpha } & \overset{\left(16\right)}{=}\frac{L_{c}}{R_{c}}. \end{array}$$

As the transient signal shall settle within a fraction ν of a period $T_{0}=2\pi /\omega_{0}$, i.e., $\tau \overset{!}{=}\nu T_{0}$, we get from (18):

(19) $$\begin{array}{c} \frac{L_{c}}{R_{c}}\overset{!}{=}\nu \cdot \frac{2\pi }{\omega_{0}}. \end{array}$$

Solving (19) for $R_{c}$ and substituting the result into (10) yields the optimal series loss resistance and resistive circuit load to achieve both fast transient settling and simultaneous impedance matching:

(20) $$\begin{array}{cc} R_{c,\mathrm{opt}} & =\frac{\omega_{0}L_{c}}{2\nu \pi }, \end{array}$$

(21) $$\begin{array}{cc} R_{L,\mathrm{opt}} & =\frac{\omega_{0}L_{c}\left(4\nu^{2}\pi^{2}+1\right)}{2\nu \pi }. \end{array}$$

Recalling from [28] that the maximum power efficiency of any inductive link system scales positively with the figure of merit

(22) $$\begin{array}{c} \xi =\frac{\omega_{0}^{2}M_{ij}^{2}}{R_{c,i}R_{c,j}}, \end{array}$$

we see that increasing the number of turns does not improve the output power and therefore signal-to-noise ratio in this high-bandwidth inductive link: a higher number of turns *N* corresponds to higher mutual inductance $M_{ij}\propto N^{2}$, but also increases the self-inductance of the coils with $L_{i}\propto N^{2}$. As the loss resistances have to scale with the inductance as given by (20) to provide fast transient settling, the effective figure of merit will remain more or less constant with *N*.

#### Design Summary

**Figure 6:**
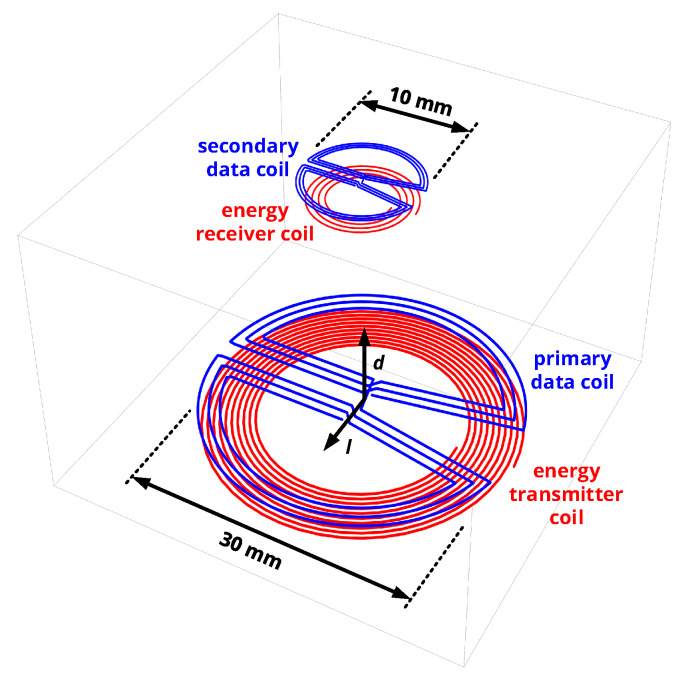
Setup of the overall coil system: A wireless power transfer link is realized by two planar spiral coils, while the data link consists of two additional butterfly-shaped coils.

The butterfly-shaped coil topology was selected for the given NFC interface design, for the reason as described in Section 3.1. A primary and secondary data coil were redesigned for $r_{b,1}=15\;\mathrm{mm}$ and $r_{b,2}=5\;\mathrm{mm}$ to match the external dimensions of the energy coils, resulting in the overall coil system of Figure 6. Using a 13.56 MHz carrier and requiring a 6.78 MHz symbol rate, we request the transient response to settle within half of a carrier cycle (ν=0.5), which implies a quality factor of the data coil of $\omega_{0}L_{c}/R_{c}=\pi$, as given by (19). To assure that the optimal load resistance $R_{L,\mathrm{par},i}$ lies in the range from 50 to 200 Ω, which is feasible to be matched to a receiving amplifier, the coils should have a self-inductance $L_{i}$ in the range from 170 to 680 nH according to (21), which can be realized with three turns each (recall the self-inductance from (7)). The coils’ pitch $p_{i}$ was arbitrarily chosen to be 0.3 mm for the primary and 0.15 mm for the secondary coil, while the trace width $w_{i}$ was dimensioned with the resistance model from [28] to obtain an ohmic coil resistance resistance well below 1/10 of the required optimal series resistance $R_{c,\mathrm{opt}}$, which is then defined by an additional lumped resistor on the circuit board of the coil. The final design parameters of the resonators are compiled in Table 1.

## 4. Analog Front-End

**Figure 7:**
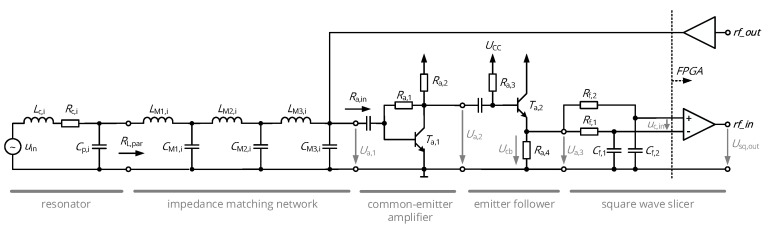
Analog front-end of the custom NFC transceiver.

Apart from the digital receiver and the NFC resonators, the data link includes an analog front-end with a receiving amplifier and a reactive network which provides both filtering of the energy carrier and impedance matching of amplifier and resonator. Its topology and connection to the low-voltage differential signaling (LVDS) comparator stage and the output buffer of the FPGA are shown in Figure 7.

### 4.1. Receiving Amplifier

The receiving amplifier is designed to increase the voltage amplitude of the incoming RF signal and to bias the LVDS stage of the FPGA. It basically consists of three subsequent stages:

- The common-emitter amplifier provides the main fraction of the voltage gain at a high input impedance. The collector feedback resistor $R_{a,1}$ allows amplifier biasing with a small number of components and limited temperature drift [31].
- The emitter follower is applied to decouple the low-power common-emitter stage from the non-negligible input capacitance of the square-wave slicer. By appropriate biasing, it adds a DC offset of approx. 1 V to the RF signal, so that the requirement for a DC biasing of the LVDS comparator in the middle of the supply range is satisfied.
- The square-wave slicer is the comparator-based circuit translating the sinusoidal RF signal to a rectangular wave showing the same OOK or PSK modulation as the input signal. Here, the comparator is fed with a low-pass filtered average of the analog signal (basically representing the DC level) through the branch of $R_{f,1}=1\;kΩ$ and $C_{f,1}=1\;\mathrm{nF}$ and a signal with fast transient behavior through $R_{f,2}=1\;kΩ$ and $C_{f,2}=6\;\mathrm{pF}$ (parasitic input capacitance), which limit the impact of noise and interference.

The circuits are optimized to by a parametric sweep of the biasing resistors $R_{a,i}$ in a small-signal simulation to yield the optimum ratio of power consumption and gain. The simulation revealed a maximum gain of 25 at a power consumption of approx. 600 μW and a resistive input impedance of 700 Ω between 1 and 20 MHz. The component values are listed in Table 2.

### 4.2. Impedance Matching

Impedance matching networks are essential to transform the amplifier’s input impedance $R_{a,\mathrm{in}}$ to the optimal load impedance of the resonators $R_{L,\mathrm{par}}$ given in Table 1. In the given application, a wideband matching must be realized due to the required high bandwidth of the communication link. Moreover, the network shall effectively suppress the energy carrier at 40.68 MHz. Both of these requirements imply a multi-stage network with low-pass characteristics, which is realized by three cascaded L networks as shown in Figure 7. According to [32], wideband matching can be established by introducing virtual intermediate resistances $R_{\mathrm{int},i}$ and by dimensioning the individual L networks in order to maintain a constant ratio *r* of input and output impedance to be matched:

(23) $$\begin{array}{c} r=\frac{R_{L,\mathrm{par},i}}{R_{\mathrm{int},1}}=\frac{R_{\mathrm{int},1}}{R_{\mathrm{int},2}}=\frac{R_{\mathrm{int},2}}{R_{a,\mathrm{in}}}. \end{array}$$

This results in the virtual intermediate resistances of

(24) $$\begin{array}{cc} R_{\mathrm{int},1,i} & ={\left(R_{L,\mathrm{par},i}\right)}^{2/3}\cdot {\left(R_{a,\mathrm{in}}\right)}^{1/3}, \end{array}$$

(25) $$\begin{array}{cc} R_{\mathrm{int},2,i} & ={\left(R_{L,\mathrm{par},i}\right)}^{1/3}\cdot {\left(R_{a,\mathrm{in}}\right)}^{2/3}. \end{array}$$

**Figure 8:**
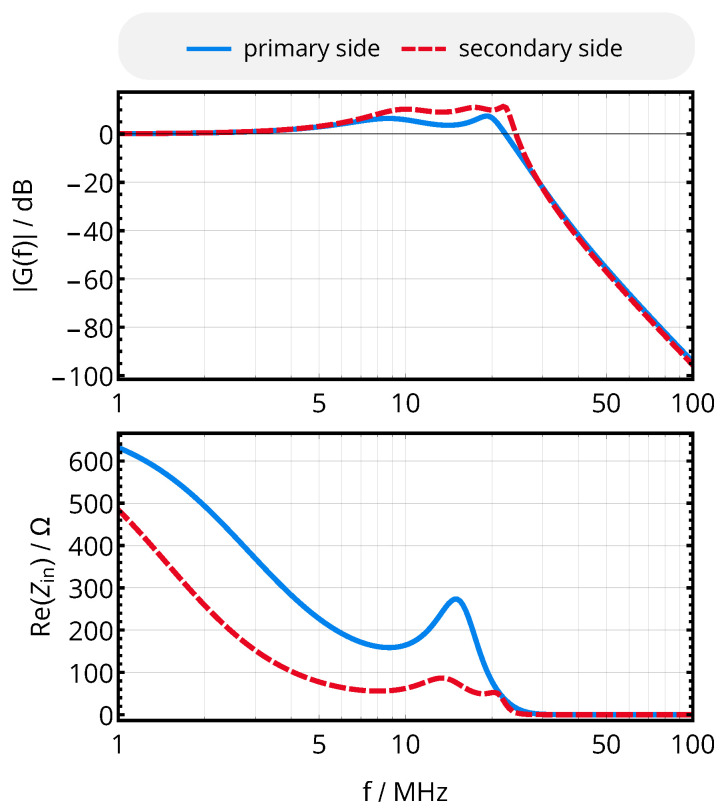
Frequency behavior of voltage transfer function and input impedance of the matching and filtering network.

As the impedance is matched over a relatively large frequency range and as the cutoff frequency of the resulting filter should be as low as possible (suppressing the power carrier at 40.68 MHz while still passing $f_{0}+f_{\mathrm{symb}}$), the nominal frequency to compute inductors and capacitors was set to 9 MHz instead of 13.56 MHz; the component values obtained from this method are given in Table 2, transfer function, and input impedance provided to the resonators are summarized in Figure 8 for the assumption of ideal components.

### 4.3. Transmitter Stage

The output buffer of the transmitter is directly connected to the node joining impedance matching network and receiving amplifier. By putting the buffer in a high-impedance state, the node can be released during signal reception, just being loaded by buffer’s parasitic capacitance. This topology was implemented for minimum circuit footprint, although energy is dissipated into receiving amplifier in the case of signal transmission. The rectangular output signal with a peak-to-peak voltage of 1.8 V, i.e., a fundamental component of ${\hat{U}}_{1}=1.15\;V$ according to a Fourier series decomposition, and the network’s input impedance of 700 Ω (conjugate complex matching to $R_{a,\mathrm{in}}$) yield an output power of the transmitter being

(26) $$\begin{array}{c} P_{\mathrm{TX}}=\frac{{\hat{U}}_{1}^{2}}{2R_{a,\mathrm{in}}}=0.94\;\mathrm{mW}. \end{array}$$

## 5. Digital Modulation and Demodulation Circuits

### 5.1. General Overview

**Figure 9:**
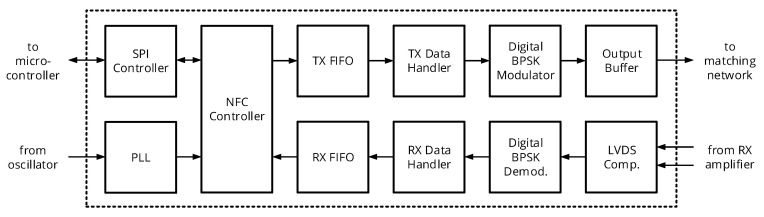
System architecture of the NFC transceiver’s digital circuitry implemented in an FPGA.

An overview of the NFC transceiver’s digital part is given in Figure 9: An NFC controller block is coordinating the data transfer, either enabling the transmit (TX) chain or the receive (RX) chain and routing data between an SPI interface and the corresponding first-in first-out (FIFO) buffer memories. The TX chain further includes a TX data handler, which builds the packets to be transmitted including carrier burst, preamble, and payload data extracted from the TX FIFO, as well as the actual modulator. The RX chain has the opposite functionality, i.e., it demodulates the data, extracts the actual payload data, and stores it into the RX FIFO. A PLL generates a base and sampling clock of 108.48 MHz from a 40.0 MHz crystal oscillator; the base clock is divided to obtain a 54.24 MHz digital core clock and a 13.56 MHz carrier clock to be used in the NFC controller block.

### 5.2. Transmitter

**Figure 10:**
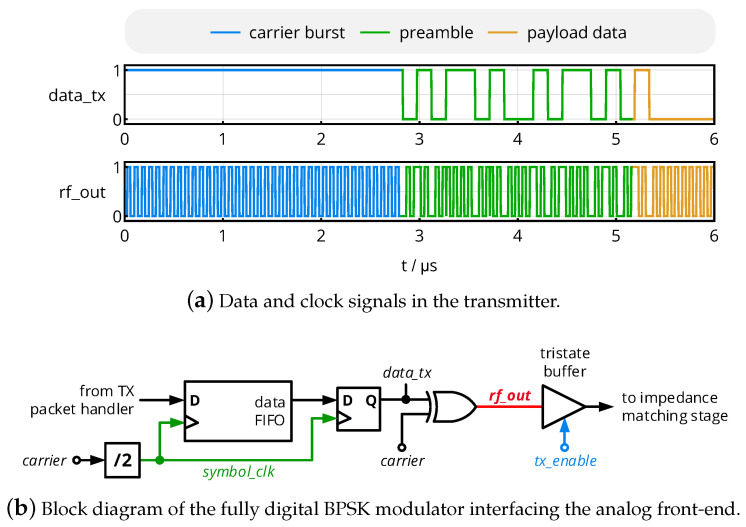
Signals and circuits of the custom NFC transmitter.

The transmitter generates a 13.56 MHz signal modulated with binary phase-shift keying (BPSK), i.e., a phase modulation with the carrier phases of 0° and 180°, at a symbol rate of 6.78 MHz. Besides the payload data, a packet will include an initial sequence as shown in Figure 10a: An initial carrier burst will allow the receiver to synchronize its local carrier, which is required for synchronous demodulation; the subsequent preamble is a data pattern containing several transitions between ’0’ and ’1’ (here: 0101101001011010), which allows the receiver to detect the beginning of the following payload data.

This data sequence is generated by the TX packet handler and fed to the BPSK modulator shown in Figure 10b. A data FIFO and a D-flipflop assure that every data bit is applied to the modulator with exactly the same length of two carrier cycles, creating the signal *data_tx*. The actual modulation is then performed by an XOR gate, which acts as a digital mixer by passing the carrier for *data_tx*=’0’ and inverts the carrier for *data_tx*=’1’. A tristate buffer either applies the modulated signal *rf_out* to the input of the impedance matching network (recall Figure 7) or switches the output to a high-impedance state in order not to load the output node in case the receiver is active.

### 5.3. Receiver

#### 5.3.1. Overview

**Figure 11:**
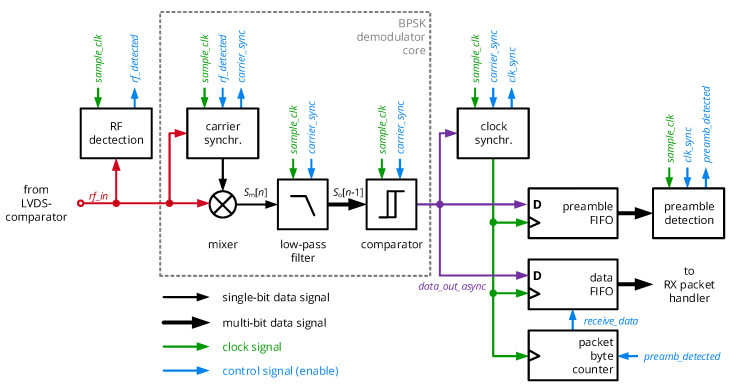
Block diagram as well as data and control signals of the fully digital BPSK demodulator.

The receiver’s digital circuit architecture is summarized in Figure 11 and composed as follows: An RF detection block checks the incoming signal from the LVDS comparator *rf_in* for RF transients and enables the subsequent circuit blocks upon detection. These include the fully custom BPSK demodulator block as a synchronous receiver, which extracts a raw data stream formally corresponding to the recovered stream of *data_tx* by the digital circuits detailed in the next subsection. A symbol clock is extracted from the raw data signal, dynamically restarting a symbol clock period at every signal transition of *data_async_out*. This clock is then used to sample *data_async_out* at the middle of a symbol clock period into the preamble FIFO, which is analyzed for the presence of the expected preamble pattern. As soon as it is detected, both data FIFO and packet byte counter are activated, so that the subsequent 64 bytes of payload data are acquired by the RX packet handler. After a fixed packet size of 64 bytes, reception is terminated and the receiver stage is reset to its initial state.

#### 5.3.2. BPSK Demodulator Core

**Figure 12:**
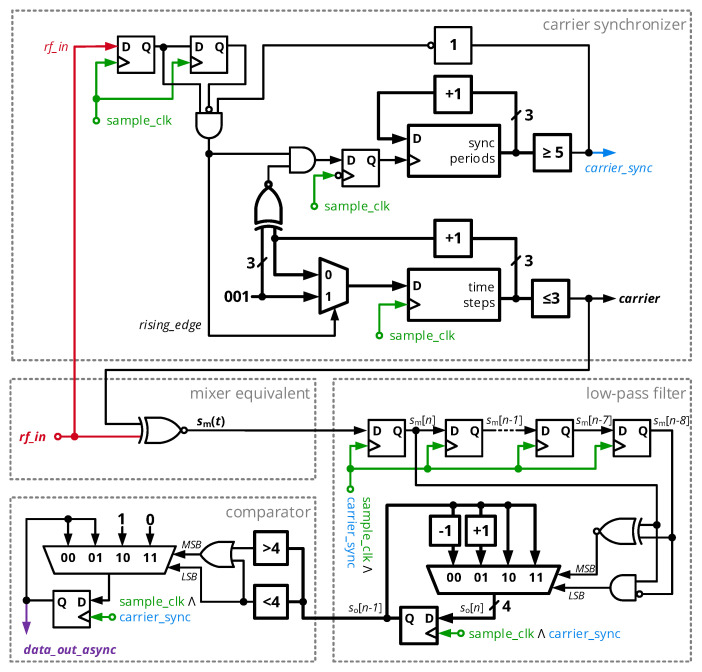
Schematic diagram of the BSPK demodulator core with sub-elements to perform carrier synchronization, down-conversion (mixing and filtering) as well as symbol mapping (comparator).

The essential element of the receiver is the BPSK demodulator core shown in Figure 12, which demodulates the rectangular wave input signal based on a compact digital circuit. It therefore replaces analog circuits such as mixers and baseband filters, which can barely be realized with a small form factor and low power consumption using discrete components, while their digital counterparts can be synthesized for a small-scale and low-power FPGA. Furthermore, it differs from fully digital receivers in the sense that it does not sample the analog signal with a multi-bit, but just a single-bit analog-to-digital converter, omitting another classical and energy intensive component of a receiver circuit. In brief, it can be categorized as a direct-RF sampling receiver with a single-bit ADC and without the demodulation path of the quadrature component [33].

Prior to down-conversion, the local carrier provided to the mixing stage must be synchronized to the incoming carrier. The initial carrier burst phase of a packet will be used for this purpose, which is processed by the carrier synchronizer stage of Figure 12. A sample clock of 108.48 MHz, i.e., eight times the carrier frequency of 13.56 MHz, acquires the input RF signal *rf_in* into a two-stage shift register in order to detect rising edges of the signal. Upon detection, a three-bit time step counter will be synchronized so that an output counter value of 0 coincides with the sampling clock period in which the rising edge of *rf_in* occurs. A decision stage maps the time step counter values 0 to 3 to a logic ’1’ of the synchronized clock signal and 4 to 7 to a logic ’0’. If the rising edge of the incoming RF signal and the anticipated period of a rising edge fall together, *rf_in* and the local carrier are synchronized for the given period. An additional circuit block counts the number of subsequent synchronized carrier periods; after more than five such cycles in series, the local carrier is considered to be synchronized and further resynchronization is suppressed until the reception is interrupted (completed packet transfer or loss of RF input signal).

The mixing process of incoming RF signal and local carrier is generally supposed to result in a large output signal of the mixer if both are in phase, while a 180° phase shift shall result in a small output signal. Considering the rectangular wave, the output signal shall be increased if both signals share the same logic level, which is implemented by an XNOR operation (with the logical negation ¬ and the XOR operator ⊕):

(27) $$\begin{array}{cc} s_{m}\left(t\right) & =\neg (s_{\mathrm{rf}\_\mathrm{in}}\left(t\right)⊕s_{\mathrm{carrier}}\left(t\right)), \end{array}$$

As this signal can show a significant amount of transient toggling during one period, its time-averaged (low-pass filtered) version should be considered as a measure of the phase difference $\phi_{\mathrm{data}}$ of the mixed signals:

(28) $$\begin{array}{c} s_{o}\left(t\right)=\frac{1}{T_{0}}\int_{0}^{T_{0}}s_{m}\left(t\right)dt=1-\left|\frac{\phi_{\mathrm{data}}}{\pi }\right|. \end{array}$$

In a discrete-time implementation, this translates to

(29) $$\begin{array}{cc} s_{o}\left[n\right] & =\sum_{i=0}^{7}s_{m}\left[n-i\right] \end{array}$$

(30) $$\begin{array}{cc} \; & =s_{o}\left[n-1\right]+s_{m}\left[n\right]-s_{m}\left[n-8\right], \end{array}$$

where the constant $1/T_{0}$ was omitted and where the equation was transformed to yield a recursive moving average filter. In the implementation, this low-pass filter is realized by a 9-bit shift register and a counter, which either increments its register value if the incoming bit $s_{m}\left[n\right]$ is ’1’ and the bit being removed from the filter $s_{m}\left[n-8\right]$ is ’0’, decrements for the opposite case and stays constant for all other cases. A subsequent decision stage maps the counters output $s_{o}\left[n\right]\in \left\{0,\cdots ,8\right\}$ to a single binary value corresponding to the decoded bit; a hysteresis avoids frequent toggling of this asynchronous data output, so that $s_{o}=4$ does not change the logic level *data_out_sync*.

In summary, the stage synchronizes to the incoming carrier and performs down-conversion and symbol mapping to yield an asynchronous data stream, which is processed by the circuitry described in Section 5.3.1.

## 6. Bit Error Rate in Single-Bit Sampling Transceivers

Comparing the given receiver to the classical topology of a mixer-based down-conversion with subsequent low-pass filtering, two characteristics are noted to be different: first of all, the input signals are rectangular waves, which results in a slightly different relation of the phase difference to be measured and the mixer’s low-pass filtered output. Secondly, the incoming analog RF signal is digitized by a comparator with hysteresis, so that a sample effectively depends on the logic level of the previous sample: The comparator disposes a certain hysteresis $U_{H}$, which toggles the output from ’0’ to ’1’ if $u_{c,\mathrm{in}}>U_{H}/2$, and evokes the transition from ’1’ to ’0’ if $u_{c,\mathrm{in}}<-U_{H}/2$. We will therefore present the mathematical foundation to allow for a numerical computation of the hysteretic single-bit sampling receiver’s bit error rate, which has not been analyzed in literature.

First of all, the input signal is defined as a sinusoidal carrier with an amplitude of ${\hat{u}}_{c,\mathrm{in}}$, a phase noise $\varphi_{n,\mathrm{TX}}\left(t\right)$ with a zero-mean Gaussian distribution and a standard deviation of $\sigma_{P,\mathrm{TX}}$ as well as an additive white noise n(t) with zero mean and a standard deviation of $\sigma_{A}$:

(31) $$\begin{array}{c} u_{c,\mathrm{in}}\left(t\right)={\hat{u}}_{c,\mathrm{in}}\sin\left(\omega t+\phi_{\mathrm{data}}\left(t\right)+\varphi_{n,\mathrm{TX}}\left(t\right)\right)+n\left(t\right). \end{array}$$

The sampling clock is also influenced by a phase noise $\varphi_{n,\mathrm{RX}}$, which is not correlated to the phase noise of the transmitted signal $\varphi_{n,\mathrm{TX}}$, so that they can be summarized into an overall phase noise term $\varphi_{n}\left(t\right)$:

(32) $$\begin{array}{cc} u_{c,\mathrm{in}}\left(t_{s}\right) & ={\hat{u}}_{c,\mathrm{in}}\sin\left(\omega t_{s}+\phi_{\mathrm{data}}\left(t_{s}\right)+\varphi_{n,\mathrm{TX}}\left(t_{s}\right)+\varphi_{n,\mathrm{RX}}\left(t_{s}\right)\right)+n\left(t_{s}\right) \end{array}$$

(33) $$\begin{array}{cc} \; & ={\hat{u}}_{c,\mathrm{in}}\sin\left(\omega t_{s}+\phi_{\mathrm{data}}\left(t_{s}\right)+\varphi_{n}\left(t_{s}\right)\right)+n\left(t_{s}\right). \end{array}$$

Here, the standard deviation of the overall phase noise $\varphi_{n}\left(t\right)$ derives as

(34) $$\begin{array}{c} \sigma_{P}=\sqrt{\sigma_{P,\mathrm{TX}}^{2}+\sigma_{P,\mathrm{RX}}^{2}}. \end{array}$$

**Figure 13:**
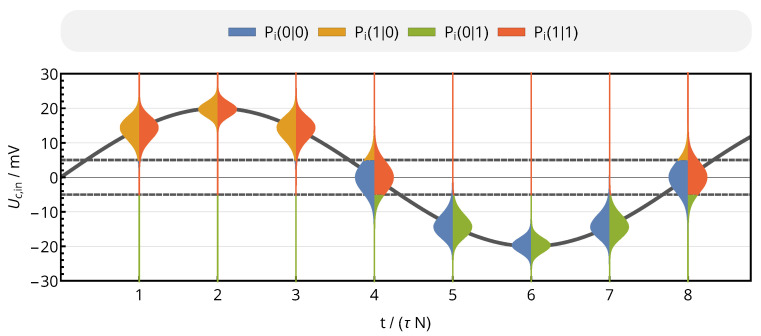
A sinusoidal RF input signal $u_{c,\mathrm{in}}\left(t\right)$ is sampled at eight points per period. As the voltage is subject to phase and amplitude noise, the acquired voltage shows a certain probability to deviate from the nominal value, expressed by a probability density function $p(u_{c,\mathrm{in}})$ being indicated by the Gaussian curves at any sampling point. Due to the hysteresis thresholds $\pm U_{H}/2$ of the comparator, illustrated by the two dashed lines, the probability of a value $x_{i}$ being sampled as ’0’ or as ’1’ is expressed by a conditional probability $P(x_{i}|x_{i-1})$ is taking the previous value $x_{i-1}$ into account. The graph is plotted for ${\hat{u}}_{c,\mathrm{in}}=20\;\mathrm{mV}$, $\sigma_{P}=2\pi \times 0.03$, $\sigma_{A}=2\;\mathrm{mV}$ and $U_{H}=10\;\mathrm{mV}$.

Hence, the standard deviations of phase noise $\sigma_{P}$ and amplitude noise $\sigma_{A}$ as well as the true momentary value of the sinusoidal input signal $u_{c,\mathrm{in}}$ determine the probability of certain voltages to be present at the input, basically defining a probability density function of the input voltage $p(u_{c,\mathrm{in}})$, which is different for every sampling point as visualized in Figure 13. In the scope of this work, $p(u_{c,\mathrm{in}})$ is computed by a Monte Carlo simulation, computing the input voltage at every sampling point according to (33) for $10^{7}$ values each.

In the next step, probability density functions and the comparator hysteresis $U_{H}$ can then be used to compute the probability of an input sample $x_{i}$ being ’0’ or ’1’: Here, it is important to remember that hysteresis requires $u_{c,\mathrm{in}}$ to rise above $U_{H}/2$ to yield a ’1’ if the previous sample was a ’0’, while it is sufficient that $u_{c,\mathrm{in}}$ stays above $-U_{H}/2$ if the previous sample was a ’1’ itself. Hence, there is no absolute probability for $x_{i}$, but a conditional probability $P(x_{i}|x_{i-1})$ that includes the previous value. To obtain this conditional probability, the probability density function must be integrated in the corresponding regions, such as illustrated in Figure 13, where the integrated areas on the left-hand side of a sampling point indicate the $P(x_{i}|0)$ (a previous sample of ’0’) and the ones on the right-hand side $P(x_{i}|1)$ (a previous sample of ’1’). In mathematical notation, the logic level $x_{i}\in \left\{0,1\right\}$ at the sampling point *i* with the sampling rate $T_{i}$ has a conditional probability of

(35) $$\begin{array}{c} P_{i}\left(x_{i}|x_{i-1}\right)=\left\{\begin{array}{cc} \int_{{\left(-1\right)}^{\left(x_{i-1}\right)}\cdot U_{H}/2}^{\infty }p\left(u_{c,\mathrm{in}}\left(t_{s}=i\cdot T_{i}\right)\right) & \mathrm{for}\;x_{i}=1\\ \int_{-\infty }^{{\left(-1\right)}^{\left(x_{i-1}\right)}\cdot U_{H}/2}p\left(u_{c,\mathrm{in}}\left(t_{s}=i\cdot T_{i}\right)\right) & \mathrm{for}\;x_{i}=0. \end{array}\right. \end{array}$$

The given approach is a simplification, as we only consider the probability density function at the sampling points, but not in the time slots in-between the sampling points. This procedure can be refined if fewer sampling points are taken into account.

**Figure 14:**
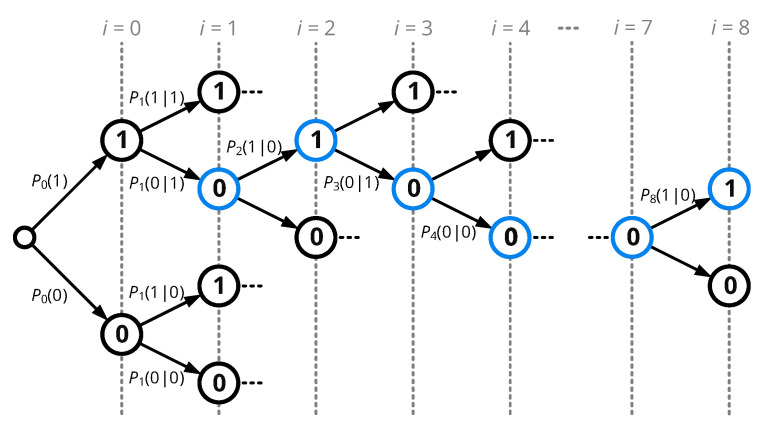
The possible sequences of samples $X_{n}$ acquired for one period can be represented as a binary tree. The transition probabilities are given by the conditional probabilities $P(x_{i}|x_{i-1})$.

Knowing the conditional probabilities of every sampling point allows for determining the probability of any possible output sequence $X_{n}=\left\{x_{1},x_{2},\cdots ,x_{N}\right\}$ of length *N*. All possible sequences can graphically be represented by the tree diagram of Figure 14: Every logical value at any sampling point can be followed by a ’0’ or a ’1’ with a certain probability, resulting in $n=2^{\left(N+1\right)}$ overall branches, as the beginning of the sequence also has two potential starting points. The probability of a specific output sequence $X_{n}=\left\{x_{1},x_{2},\cdots ,x_{N}\right\}$ can then be calculated by multiplying the conditional probabilities along its path, and by adding the results of the path starting with an initial ’0’ and that starting with a ’1’:

(36) $$\begin{array}{cc} P_{\mathrm{seq}}\left(\left\{x_{1},x_{2},\cdots ,x_{N}\right\}\right) & =P\left(\left\{0,x_{1},x_{2},\cdots ,x_{N}\right\}\right)+P\left(\left\{1,x_{1},x_{2},\cdots ,x_{N}\right\}\right)\\ \; & =P_{0}\left(x_{0}\right)\cdot \prod_{i=1}^{N}P_{i}\left(x_{i}|x_{i-1}\right){|}_{x_{0}=0}+P_{0}\left(x_{0}\right)\cdot \prod_{i=1}^{N}P_{i}\left(x_{i}|x_{i-1}\right){|}_{x_{0}=1} \end{array}$$

Here, we assume that the transition from the previous symbol is instantaneous and that ’0’ and ’1’ are equally likely, which results in $P_{0}\left(x_{0}\right)=0.5$.

Knowing the probability of all sequences allows for selecting the carrier samples $C_{\mathrm{seq}}=\left\{c_{1},c_{2},\cdots ,c_{N}\right\}$ to be synchronized on, the cyclic permutation of the sampling sequence {1,1,1,1,0,0,0,0} having the largest value of $P_{\mathrm{seq}}$.

Recalling the XNOR operation from (27) and the summation of eight samples from (29) together with the carrier sequence $C_{\mathrm{seq}}$, every input sequence $X_{n}$ can be mapped to a certain demodulator output value $s_{o}\in \left\{0,\cdots ,8\right\}$:

(37) $$\begin{array}{c} s_{o}\left(\left\{x_{1},x_{2},\cdots ,x_{N}\right\}\right)=\sum_{i=1}^{8}s_{m,i}=\sum_{i=1}^{8}\neg \left(x_{i}⊕c_{i}\right). \end{array}$$

Further defining a set $X_{s}$ that includes all sequences $X_{n}$ resulting in an output sum of $s_{o}$ with

(38) $$\begin{array}{c} X_{s}=\left\{X_{n}\;|\;s_{o}\left(X_{n}\right)=s\right\}, \end{array}$$

we can express the probability of a certain output sum $s_{o}$ by adding up the sequence probabilities $P_{\mathrm{seq}}$ that lead to the given output value:

(39) $$\begin{array}{c} P_{\mathrm{out}}\left(s\right)=\sum_{X_{n}\in X_{s}}P_{\mathrm{seq}}\left(X_{n}\right) \end{array}$$

With the symbol mapper defined in Section 5.3.2, an output of $s_{o}>4$ is mapped to a symbol value ’1’, and $s_{o}<4$ to ’0’, while $s_{o}=4$ does not evoke a change in the symbol value, statistically leading to a bit error in half of the cases. As we defined the carrier sequence $C_{\mathrm{seq}}$ to be in-phase with the incoming data sequence, a bit error occurs if a symbol of ’0’ is detected, so that the bit error rate is given by:

(40) $$\begin{array}{c} \mathrm{BER}=\left(\sum_{s=1}^{3}P_{\mathrm{out}}\left(s\right)\right)+\frac{1}{2}\cdot P_{\mathrm{out}}\left(4\right), \end{array}$$

In summary, the bit error rate of a single-bit sampling receiver can numerically be determined by computing the probability density function at the sampling points for a given set of noise parameters, which allows for deriving conditional probabilities from (35), sequence probabilities from (36), output sum probabilities from (39), and finally the bit error rate from (40).

## 7. System Realization

**Figure 15:**
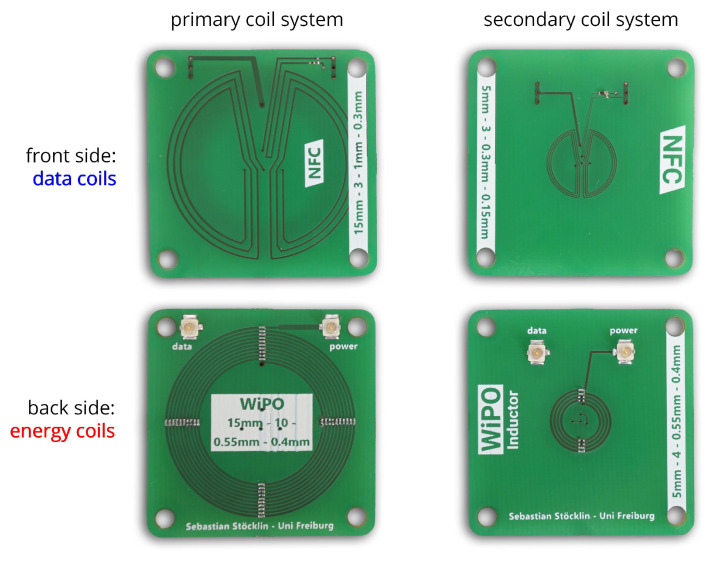
Prototype coil systems manufactured on an FR4 substrate. The data coil structures include resistive and capacitive tuning elements, the energy coils are realized as capacitively segmented coils.

The data coils specified in Table 1 were implemented on a commercially available two-layer printed circuit board (PCB) of 1.6 mm thickness as shown in Figure 15, together with the optimized and segmented data coils from [28]. The series resistors $R_{c,\mathrm{opt},i}$ and parallel capacitors $C_{\mathrm{par},i}$ were included as lumped elements with a component size of 0201 to yield the values of Table 1.

**Figure 16:**
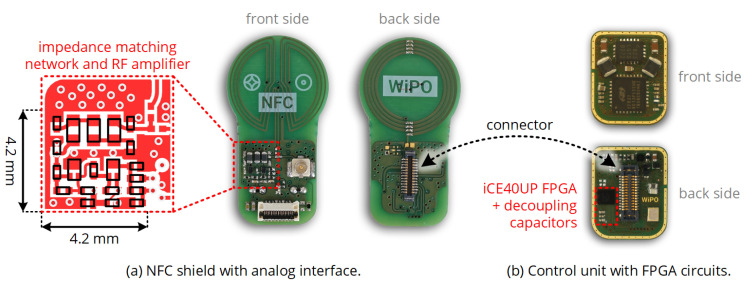
Prototype circuits implementing the custom NFC transceiver: A shield including the analog front-end and the coil connectors is mounted on a general purpose control unit including DC/DC converters, an RF microcontroller, and a low-power FPGA. The net area required for the NFC transceiver is enclosed by the colored contours.

The analog front-end was implemented on a four-layer PCB shield shown in Figure 16: The amplifier consists out of two *Infineon BFR840L3RHESD* NPN transistors and 0201 SMD resistors, the impedance matching and filtering network includes shielded *TDK MLF1608A* 0603 inductors and ceramic 0201 capacitors with C0G dielectric. The PCB also includes a 7 mm butterfly-shaped data coil, which was not used in the context of this work.

The shield was connected to a miniaturized control unit (also see Figure 16) carrying a *Lattice iCE40UP4K* FPGA and a *Silicon Labs EFR32BG13P* microcontroller, along with various voltage regulators providing a 1.8 V supply for the FPGA’s I/Os and the receiving amplifier and a 1.2 V supply for the FPGA’s digital core. The digital transceiver unit was implemented by as a behavioral model following the description of Section 5 and synthesized by the *Lattice Radiant* toolchain. Moreover, the microcontroller was configured to act as bootloader and test data source for the FPGA circuits.

## 8. Results

### 8.1. Coil Parameters

**Figure 17:**
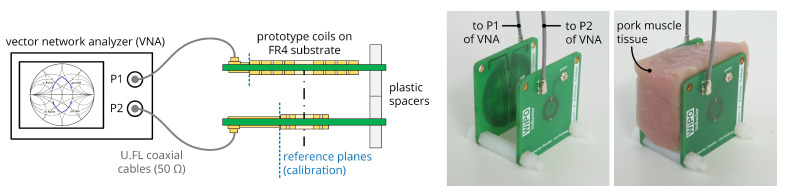
Measurement setup to determine the S-parameters of the coil systems in air and pork tissue environment.

In the first step of experimental evaluation, the coil parameters were determined by the measurement setup shown in Figure 17 and by the procedure detailed in [28], i.e., the S-parameters of the data coil link were acquired with a vector network analyzer, converted to Z-parameters and subsequently to self-inductance Li=Im(Zii)/ω0 and mutual inductance Mij=Im(Zij)/ω0. The measured coil and coupling parameters for various coil separation distances $d_{34}$ acquired in air are listed in Table 3 together with the computed values according to (6) and (7). With a maximum relative deviation of 5.2 %, simulated and measured values are in close agreement.

**Figure 18:**
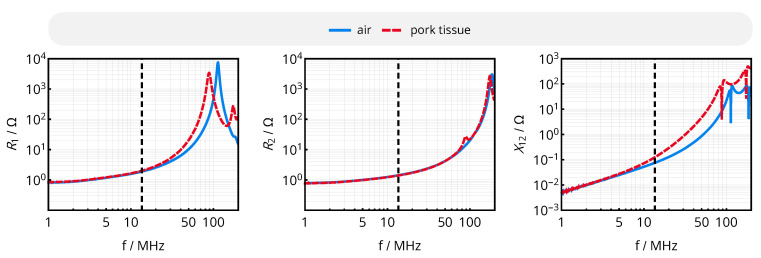
Frequency behavior of the effective coil resistances $R_{i}$ and the mutual reactance $X_{12}$ for the given data coil system at a coil separation distance of $d_{34}=20\;\mathrm{mm}$ in air and pork muscle tissue.

With the same setup, the coils were analyzed in the environment of homogenous pork muscle tissue. The frequency behavior of the effective coil resistances Ri=Re(Zii) and the mutual reactance X12=Im(Z12) are compared to the characteristics of an air coil system in Figure 18: While the resonance frequencies of both data coils being subject to tissue are decreased and the width of the resonance peak is increased due to additional losses, the parameters show only minor deviations at the target operational frequency of 13.56 MHz. This can be attributed to the low number of turns of the presented data coils, and must be reevaluated for deviating designs with increased overall dimensions, decreased trace pitch and increased number of turns. Consequently, the given system implementation shows comparable results in both media, and is further evaluated in air for the sake of simplicity.

### 8.2. Output Signals

**Figure 19:**
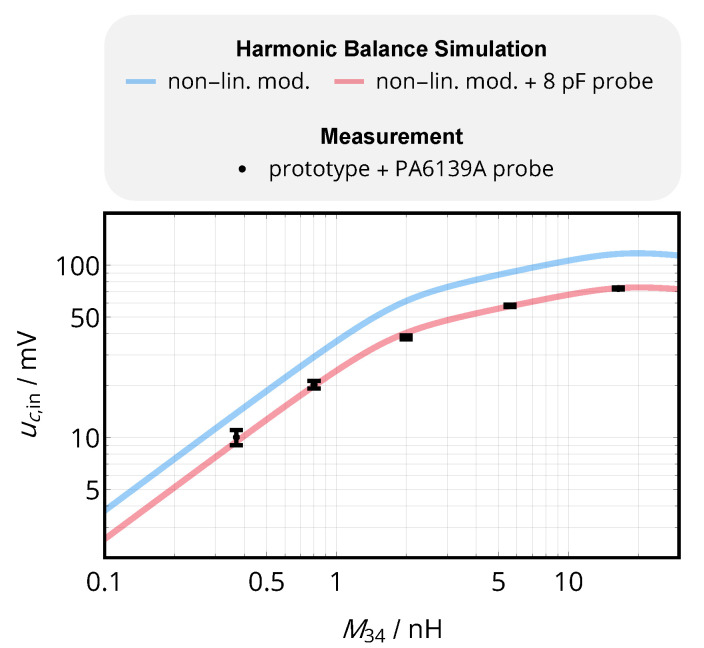
Voltage at the comparator of the receiving stage $u_{c,\mathrm{in}}$ versus mutual inductance $M_{34}$ of the data coils (in air). The simulated data was provided by a Harmonic Balance simulation using *Keysight Advanced Design System 2016*, the measurement was performed with the prototype circuits presented above.

In the next step, the input voltage of the receiver’s comparator $u_{c,\mathrm{in}}$, i.e., the output voltage of the complete wireless link including the analog interfaces, is analyzed for various levels of the mutual inductance $M_{34}$, corresponding to variations in coil distance and alignment. The voltage is simulated with the harmonic balance algorithm implemented in *Keysight Advanced Design System 2016*. A SPICE model of the *BFR840* transistor was used to simulate the receiving amplifier and the FPGA’s output stage was modeled by an output resistance of $R_{\mathrm{TX},\mathrm{out}}=300\;$ Ω and a voltage source alternating between 0 V and $U_{\mathrm{DD}}=1.8\;V$; the measurement probe was modeled by a 8 pF capacitor to ground. Data were also acquired from on the prototype implementation presented above, using a *Tektronix MSO4104* oscilloscope and a *PA6139A* probe. As shown in Figure 19, the comparator input voltage can be reproduced by the numerical simulation and is highly sensitive to capacitive loading, so that a simulative de-embedding of the probe’s capacitance is required (see blue curve). The output voltage is subject to compression for $M_{34}>2\;\mathrm{nH}$ due to the nonlinear behavior of the amplifier and drops below 60 mV (which will later to be determined as the sensitivity threshold) at $M_{34}\approx 2\;\mathrm{nH}$, which corresponds to a distance of 15 mm.

**Figure 20:**
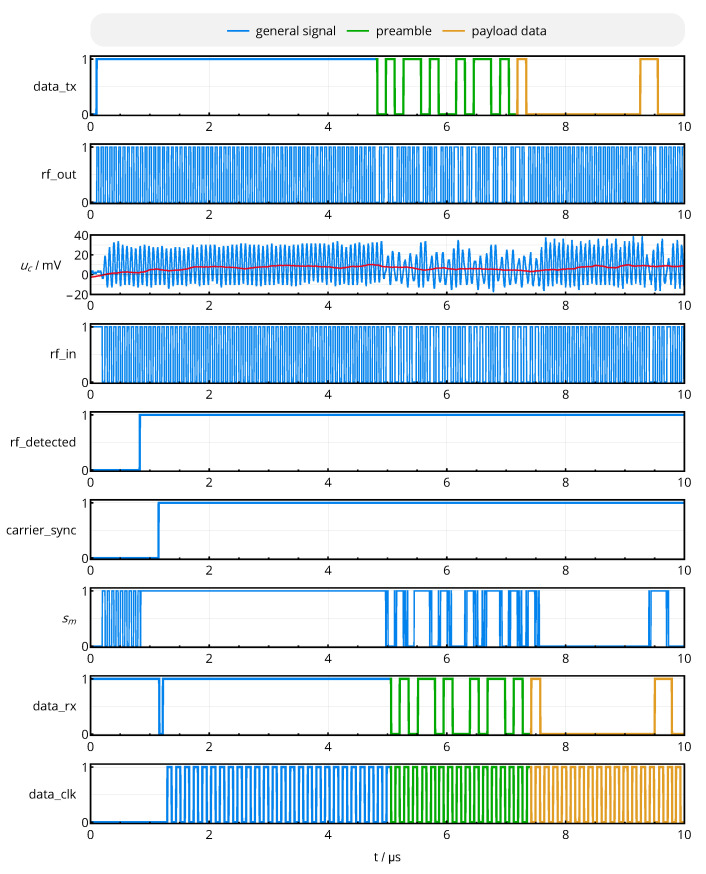
Transient behavior of important analog and digital signals within the near-field interface.

A snapshot of the link’s most important intermediate and output signals is shown in Figure 20, which were simultaneously acquired by a *Saleae Logic Pro 8* multi-channel logic analyzer and a *Rigol MSO5074* oscilloscope: The time sequence descriptively shows how the outgoing data stream is translated into a BPSK-modulated rectangular wave, which is then transmitted over the analog channel and recreated by the receiver’s comparator. After RF detection and synchronization, the mixer’s output signal $s_{m}$ is low-pass filtered to obtain the received data sequence *data_rx* and recovered clock *data_clk*, which corresponds to the incoming data sequence. Preamble and payload data are highlighted to show the identity of *data_tx* and *data_rx* and to prove the operability of the link.

### 8.3. Resources

**Figure 21:**
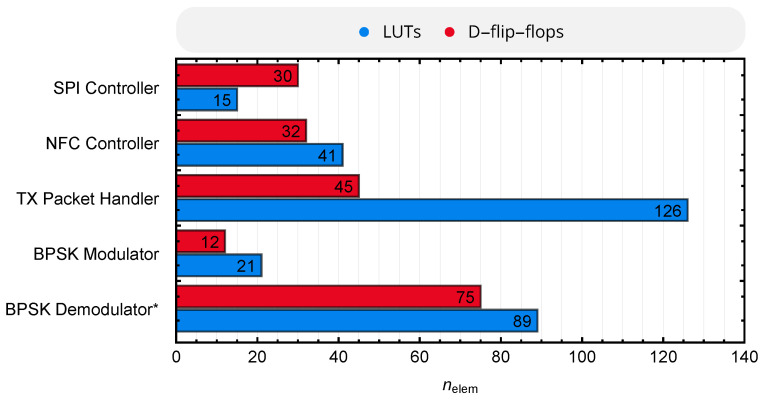
Required resources of the NFC transceiver implemented in a *Lattice iCE40UP5K* FPGA. ${}^{*}$ The BPSK Demodulator also includes components of the RX Packet Handler.

Being mainly realized in form of digital circuit blocks implemented in an FPGA, the number of look-up tables (LUTs) and D-flip-flops required to realize the transceiver functionality are of major interest. These resources are extracted from the *Lattice Radiant* toolchain of version 1.1 with the optimization goal ’area’ and summarized in Figure 21, divided among the circuit blocks of Figure 9. TX packet handler and BPSK demodulator naturally require most of the components; as the BPSK demodulator includes various shift registers for sampling and storage, the number of required D-flip-flops is particularly high. The complete digital circuit requires 373 LUTs and 196 D-flip-flops.

In terms of PCB footprint, the analog front-end and the FPGA circuit cover a net single-sided PCB area of 33 ${\mathrm{mm}}^{2}$.

### 8.4. Power Consumption

In order to account for power consumption, the current of the 1.2 V and 1.8 V supply rails of the FPGA as well as the amplifier supply current were measured both in transmitting mode with continuous data streaming as well as in receiving mode. Currents, voltages, and resulting power consumption levels are listed in Table 4. The digital circuit blocks not required for the particular transceiver functionality were disabled by clock gating. In total, the transceiver consumes 4.3 mW in transmitting mode and 2.5 mW in receiving mode. Combined with a raw data rate of 6.78 MBit/s, this results in an energy consumption of 646 pJ/bit for the transmitter and of 364 pJ/bit for the receiver.

### 8.5. Bit Error Rate

**Figure 22:**
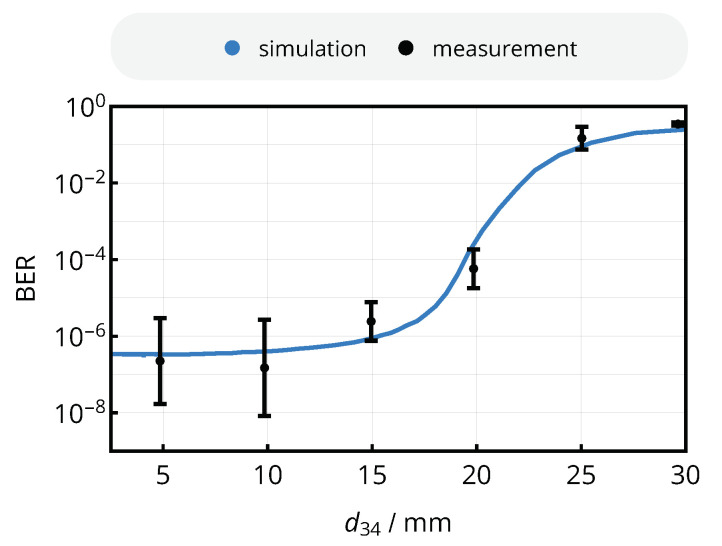
Bit error rate versus coil separation distance $d_{34}$ in simulation and measurement to characterize the complete link. The simulated data are obtained from the mathematical description of this work and the parameters $\sigma_{P}=2\pi \times 0.04$, $\sigma_{A}=8\;\mathrm{mV}$ and $U_{H}=20\;\mathrm{mV}$; the experimental values of the BER include the measurement with five different receiver PCBs and $1.2\times 10^{8}$ bits of a PRBS-15 sequence each.

Ultimately, the bit error rate of the link was measured for six different coil separation distances from 5 mm to 30 mm with a step size of 5 mm with the coil system of Figure 17; the circuits of Figure 16 are connected as transmitter and receiver. For each measurement configuration, $3\times 10^{5}$ packets with a length of 64 bytes each ($1.6\times 10^{8}$ bits), composed according to a pseudo-random bit sequence (PRBS-15), were transmitted and analyzed using five different receiver board samples. The input data were generated and injected by the microcontroller circuit of the test platform; transmitted and received data including their clock signals were captured and decoded by a *Saleae Logic Pro 8* logic analyzer and a custom Python script comparing outgoing and incoming data. The bit error rate was also determined theoretically: For every coil separation distance, the mutual inductance was computed from (6), the comparator input voltage was obtained from the relation of Figure 19, and the BER was finally derived from the mathematical model presented in Section 6. The phase noise parameter $\sigma_{P}=2\pi \times 0.04$ was acquired from the single sideband spectrum of the FPGA’s internal 13.56 MHz clock signal routed to an FPGA output, recorded by a spectrum analyzer and processed according to ([34], p. 46); the amplitude noise metric $\sigma_{A}=8\;\mathrm{mV}$ was acquired from the spectral power density of the signal $u_{c,\mathrm{in}}$ recorded by an oscilloscope without any incoming data signal. The results of both measurement and simulation are shown in Figure 22: The NFC interfaces achieves a bit error rate as low as

(41) $$\begin{array}{c} \mathrm{BER}\left(d=10\;\mathrm{mm}\right)\approx 2\times 10^{-7} \end{array}$$

for coil separation distances of up to 10 mm, and converges to the theoretical maximum of 0.5 at 30 mm. For a hysteresis of $U_{H}=20\;\mathrm{mV}$, the simulation delivers a suitable estimation of the system’s bit-error rate for all coil distances.

**Figure 23:**
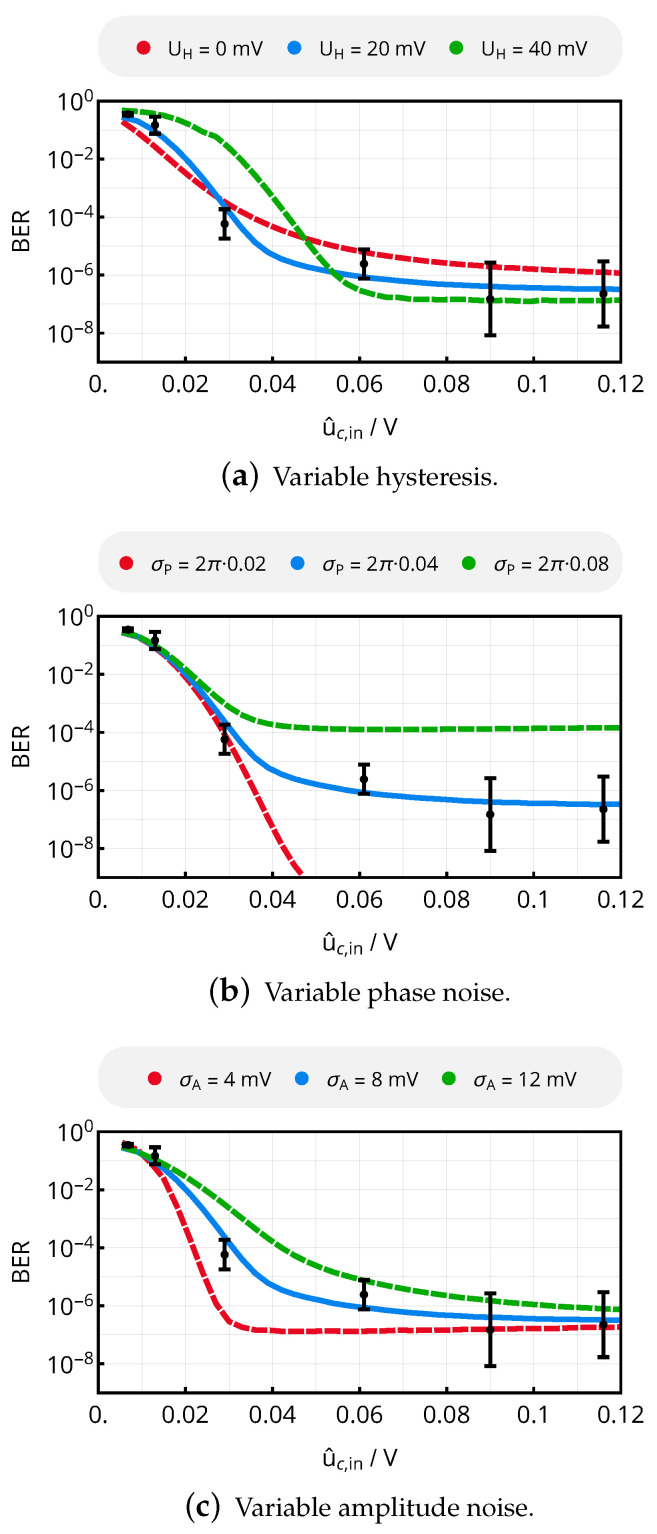
BER vs. comparator input voltage for varying hysteresis, phase noise or amplitude noise parameters according to the model of Section 6. The nominal values of the blue curves are $\sigma_{P}=2\pi \times 0.04$, $\sigma_{A}=8\;\mathrm{mV}$ and $U_{H}=20\;\mathrm{mV}$. The measurement results of Figure 22 were related to the corresponding input voltage ${\hat{u}}_{\mathrm{in}}$ and are shown in black color as a reference for the nominal noise parameters.

To characterize the receiver only, BER was analyzed versus the input voltage of the comparator stage. Here, hysteresis $U_{H}$ and noise parameters $\sigma_{P}$ and $\sigma_{A}$ are varied from the nominal values in simulation to show the impact of further system optimization. From the results shown in Figure 23, it can be noted that

- a smaller hysteresis increases BER for large signals as noise can rather lead to the incorrect detection of the input signals, but lowers BER for smaller amplitudes, as a smaller hysteresis allows small signals to exceed the toggling thresholds.
- the best-case bit error rate for large signal amplitudes is limited by the standard deviation of phase noise.
- the size of amplitude noise determines the BER’s behavior in the transition region according to the behavior of the signal-to-noise ratio.

A reduction of phase noise by appropriate modification of the oscillators and a reduction of amplitude noise by improved supply decoupling could therefore help to improve the system’s reliability even further. Apart from improvements of the physical link, error-detecting codes such as cyclic redundancy checks or even error-correcting codes [35] can be utilized to improve the effective BER, but at the cost of additional data overhead reducing the effective data rate.

Defining the input sensitivity level to yield a BER smaller than $10^{-6}$, which is given for ${\hat{u}}_{c,\mathrm{in}}\approx 60\;\mathrm{mV}$, and considering an amplifier gain of $G_{\mathrm{rec}}=25$ and an amplifier input resistance of $R_{\mathrm{in},\mathrm{rec}}=700\;$ Ω, the receiver’s input sensitivity can finally be determined to be

(42) $$\begin{array}{c} S=P_{\mathrm{in}}\left(\mathrm{BER}<10^{-6}\right)=\frac{{\left({\hat{u}}_{c,\mathrm{in}}\left(\mathrm{BER}<10^{-6}\right)\right)}^{2}}{2\cdot R_{\mathrm{in},\mathrm{rec}}\cdot G_{\mathrm{rec}}^{2}}\approx 9.1\;\mathrm{nW}\approx -54\;\mathrm{dBm}. \end{array}$$

### 8.6. Coexistence with Wireless Power Transfer

To evaluate the concept of the low-interference coil design, the mutual inductance of energy and data coils of the same substrate were measured with the method described in Section 8.1. The acquired values at the operational frequency of the power transfer link at 40.68 MHz are $M_{13}=2.1\;\mathrm{nH}$ and $M_{24}=2.8\;\mathrm{nH}$, being approximately as large as the data coils’ mutual inductance at a distance of 15 mm. The resulting coupling factors $k_{ij}=M_{ij}/\sqrt{L_{i}L_{j}}$ are $k_{13}\approx 1.6\times 10^{-3}$ and $k_{24}\approx 1.6\times 10^{-2}$, so that the on-substrate coils are subject to weak coupling.

**Figure 24:**
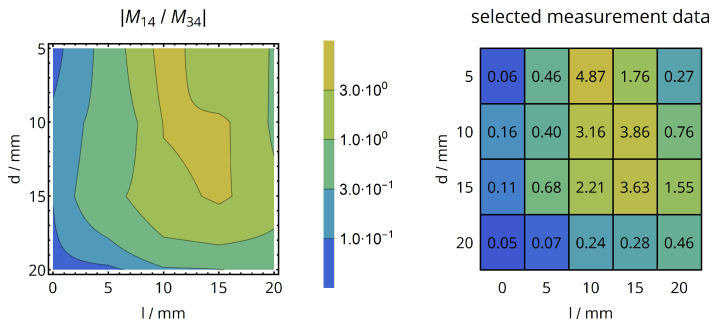
Cross-coupling analysis of data and energy coils in pork tissue environment: The ratio of $M_{14}\left(40.68\;\mathrm{MHz}\right)$ and $M_{34}\left(13.56\;\mathrm{MHz}\right)$ is shown versus coil distance *d* and lateral misalignment *l*, the measurement values on a grid with 5 mm resolution are shown as a numerical reference.

The isolation of energy and data coil of different devices, exemplarily determined for the primary energy coil and the secondary data coil, was also analyzed by acquiring the ratio of mutual inductances: The measure of interference $M_{14}\left(40.68\;\mathrm{MHz}\right)$ was divided by the measure of data link coupling $M_{34}\left(13.56\;\mathrm{MHz}\right)$. As capacitive and resonant effects might be more pronounced for a lossy dielectric environment and the higher operational frequency of the energy link (40.68 MHz), the cross-coupling analysis was performed in pork muscle tissue. The results are shown in Figure 24: For axial alignment, $M_{14}$ is more than 10 times smaller than $M_{34}$, which validates the operational principle of the butterfly-shaped coils even in lossy dielectric media.

**Figure 25:**
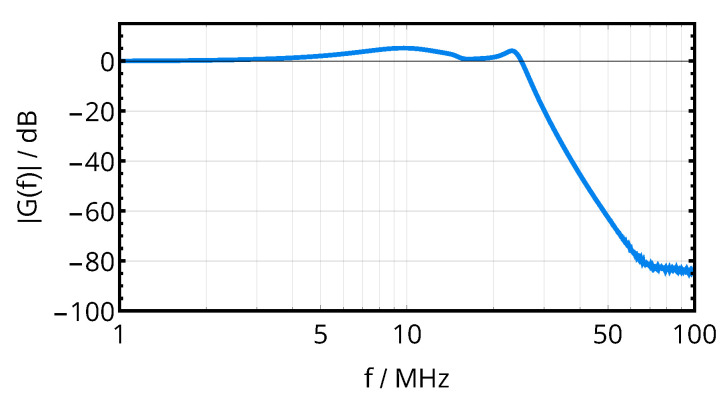
Measured voltage transfer function of the primary impedance matching network.

Moreover, the voltage transfer function of the matching network was derived from a two-port S-parameter measurement. The result of Figure 25 indicates a suppression of the energy carrier by 50 dB with respect to the data carrier, providing additional isolation.

Finally, the bit error rate resulting from the combined effect of butterfly-shaped coils and filter network was experimentally determined for a data coil separation distance of 10 mm: A class E driver stage was driving the primary energy coil with a 40.68 MHz energy carrier at a power level of 100 mW. Data were sent from the secondary data coil to the primary data coil, so that the energy transmitter was in close proximity to the data receiver. With the methods of Section 8.5, a bit error rate of $3\times 10^{-5}$ was measured, which is one order of magnitude higher than the result of the interference-free operation (cf. Figure 22), but still tolerable in a practical implementation.

## 9. Discussion

In this paper, we analyzed and experimentally verified a series of design strategies for near-field communication interfaces to enable an operation with high data rate, low power consumption, small footprint, and resilience against interference from wireless power transfer links:

First, it was shown that data coil topologies can be optimized for minimal inductive coupling to wireless power transfer coils situated on the same substrate. While the C-shaped coil performs well for a lateral offset of primary and secondary coil systems, the butterfly-shaped coil combines the advantage of small interference to the complete energy coil system for axial alignment (even for the coil not being on the same substrate) and the advantage of not having zeros of data coil coupling for an axial alignment. This is also supported by the experimental data, which showed that computed and realized self-inductances and mutual inductances are in close agreement, with a maximum relative deviation of about 5%. Moreover, the selective coupling of the data coils on the one hand and the limited mutual inductance of data and energy coils on the other hand even persist in dielectric media, as long as the coils show minor detuning due to a low number of turns.

Complementing the design of the resonators, the optimal resonator tuning obtained from the mathematical analysis and the analog front-end successfully translated into a design with the desired characteristics of fast transient settling, as shown by the acquired link signals of Figure 20. The voltage amplitudes at the receiver (cf. Figure 19) and the energy carrier suppression of the impedance matching network (cf. Figure 8 and Figure 25) are in close agreement to the simulated designs.

Secondly, the proposed concept of a single-bit sampling digital transceiver architecture was successfully evaluated: Here, the conversion of an analog sinusoidal signal to a rectangular wave by a comparator stage, the clock synchronization by a custom and minimalistic digital counter circuit, the down-conversion to baseband by a single XNOR gate, the subsequent low-pass filtering by digital moving average filter, and the symbol mapping using a simple decision stage resulted in a series of favorable characteristics: The digital design can be synthesized with less than 400 look-up tables and 200 D-flip-flops, so that it can be programmed into the smallest of today’s commercially available FPGA circuits. Although the internal sampling clocks are as high as 108.48 MHz, the power consumption is limited to 4.4 mW for the transmitter and 2.5 mW for the receiver, which yields a normalized energy consumption of 646 pJ/bit for the transmitter and of 364 pJ/bit for the receiver at a data rate of 6.78 MBit/s. Due to the mostly-digital implementation with the FPGA and the compact analog front-end, a net PCB footprint of 33 mm${}^{2}$ is achieved, which is well below the initial request of 100 mm${}^{2}$.

The synchronous demodulation and the sampling of eight points per carrier period result in a bit-error rate as low as $2\times 10^{-7}$ for coil separation distances of up to 10 mm. The measured BER levels are in the same order of magnitude as the predictions of the proposed simulation concept, which takes the hysteretic behavior of the comparator stage into account.

These given performance metrics are compared to state-of-the-art high-speed transceivers in Table 5: Here, the custom transceiver of this work provides an energy consumption per bit which is approximately 10 times lower than that of commercial NFC devices. At the same time, it outperforms the devices with respect to the maximum data rate, which is available for both transmitter and receiver. Although the data rates of transceivers with pulse-harmonic modulation exceed the given concept, it must be noted that these are highly susceptible to interference, especially to strong signals from an additional wireless power transfer link. The energy consumption of the given transceiver is within the same order of magnitude, but also includes data and packet handling circuits as well as the energy overhead stemming from the FPGA’s general purpose circuits.

## 10. Conclusions

In summary, very high bit rate near-field interfaces with low power consumption and low bit-error rate can be realized with a minimal analog front-end and a mostly-digital single-bit sampling transceiver, even with commercially available circuit components. Combined with a figure-8 data coil design that minimizes interference to nearby regular planar spiral coils and an impedance matching network suppressing interference signals in known frequency bands, a cointegration of high-speed data communication with wireless power transfer interfaces is possible, so that future generations of biomedical sensor systems can be enabled by the presented concepts and circuit topologies.

## Figures and Tables

**Table 1:** Dimensions and electrical parameters of the designed resonators.

*i*	$r_{b,i}$	$o_{b,i}$	$\alpha_{b,i}$	$p_{i}$	$w_{i}$	$N_{i}$	$s_{b,i}$	$L_{i}$	$R_{c,\mathrm{opt},i}$	$R_{L,\mathrm{par},i}$	$C_{p,i}$
3	15 mm	2 mm	10	1 mm	0.3 mm	3	0.16 mm	651 nH	17.7	192	192 pF
4	0.55 mm	0.6 mm	10	0.3 mm	0.15 mm	3	0.14 mm	263 nH	7.1	77	475 pF

**Table 2:** Component values of the analog front-end.

	Impedance Matching							Receiving Amplifier				
*i*	$R_{L,\mathrm{par},i}$	$L_{M1}$	$L_{M2}$	$L_{M3}$	$C_{M1}$	$C_{M2}$	$C_{M3}$	$R_{a,\mathrm{in}}$	$R_{a,1}$	$R_{a,2}$	$R_{a,3}$	$R_{a,4}$
3	192 Ω	2.5 μH	5.0 μH	4.8 μH	44 pF	30 pF	12 pF	700 Ω	20 kΩ	5 kΩ	100 kΩ	10 kΩ
4	77 Ω	1.2 μH	2.9 μH	5.0 μH	94 pF	44 pF	17 pF	700 Ω	20 kΩ	5 kΩ	100 kΩ	10 kΩ

**Table 3:** Inductance values in simulation and measurement (in air).

Param.	$L_{3}$	$L_{4}$	$M_{34}$			
$d_{34}$	-	-	5 mm	10 mm	15 mm	20 mm
sim.	650 nH	260 nH	16.4 nH	5.46 nH	2.0 nH	0.81 nH
meas.	680 nH	270 nH	15.8 nH	5.50 nH	2.02 nH	0.85 nH
dev.	4.6 %	3.8 %	3.6 %	0.8 %	1.0 %	5.2 %

**Table 4:** Voltages, currents, and power consumption levels of the transmitter and receiver circuits.

Parameter	Transmitting Mode	Receiving Mode
$U_{\mathrm{core}}$	1.2 V	1.2 V
$I_{\mathrm{core}}$	1400 μA	1360 μA
$P_{\mathrm{core}}$	1680 μW	1630 μW
$U_{I/O}$	1.8 V	1.8 V
$I_{I/O}$	1500 μA	190 μA
$P_{I/O}$	2700 μW	340 μW
$U_{\mathrm{RX},\mathrm{amp}}$	0 V (gated)	1.8 V
$I_{\mathrm{RX},\mathrm{amp}}$	0 μA	280 μA
$P_{\mathrm{RX},\mathrm{amp}}$	0 μW	500 μW
$P_{\mathrm{total}}$	**4380 μW**	**2470 μW**

**Table 5:** Literature overview of NFC transceivers with support for very high bit rates.

Device/Author	Technology	Mod.	$f_{c}\;\mathrm{in}\;\mathrm{MHz}$	Data Rate ${}^{1}$ in MBit/s	BER	$E_{\mathrm{bit}}$${}^{1}$ in pJ/bit	Ref.
commercial transceivers							
ST ST25R3911B	CMOS	OOK	13.56	6.78/3.34	N/A	N/A/6620	[11]
NXP NxH2281	CMOS	OOK	10.579	0.596	N/A	5700/5700	[36]
custom transceivers in academic research							
Mandal et al.	CMOS	LSK	25.0	2.8	$2.0\times 10^{-6}$	36/893 ${}^{2}$	[12]
Simard et al.	CMOS	4-PSK	13.56	4.16	$2.0\times 10^{-6}$	N/A	[13]
Kiani et al.	CMOS	PHM	66.6	20.0	$8.0\times 10^{-8}$	180/12.5 ${}^{2}$	[15]
Kiani et al.	CMOS	PHM	13.56	13.56	$4.3\times 10^{-7}$	960/162 ${}^{2}$	[16]
Schormans et al.	CMOS	SQuirM	205.5	50.4	$4.5\times 10^{-10}$	44/100 ${}^{2}$	[18]
this work	discrete + FPGA	2-PSK	13.56	6.78	$2.0\times 10^{-7}$	646/364	-

${}^{1}$ Transmitter/receiver values; ${}^{2}$ Only modulator/demodulator without packet handlers; LSK: Load Shift Keying, PHM: Pulse Harmonic Modulation, SQuirM: Short-Range Quality Factor Modulation.
